# Targeting ferroptosis: a new therapeutic opportunity for kidney diseases

**DOI:** 10.3389/fimmu.2024.1435139

**Published:** 2024-07-03

**Authors:** Zhiyong Long, Yanfang Luo, Min Yu, Xiaoyan Wang, Liuting Zeng, Kailin Yang

**Affiliations:** ^1^ Department of Physical Medicine and Rehabilitation, The Affiliated Panyu Central Hospital of Guangzhou Medical University, Guangzhou, China; ^2^ Department of Nephrology, The Central Hospital of Shaoyang, Shaoyang, Hunan, China; ^3^ Department of Rheumatology and Clinical Immunology, Peking Union Medical College Hospital, Chinese Academy of Medical Sciences & Peking Union Medical College, National Clinical Research Center for Dermatologic and Immunologic Diseases (NCRC-DID), Key Laboratory of Rheumatology and Clinical Immunology, Ministry of Education, Beijing, China; ^4^ Key Laboratory of Hunan Province for Integrated Traditional Chinese and Western Medicine on Prevention and Treatment of Cardio-Cerebral Diseases, Hunan University of Chinese Medicine, Changsha, China

**Keywords:** ferroptosis, iron metabolism, acute kidney disease, chronic kidney disease, natural compounds

## Abstract

Ferroptosis is a form of non-apoptotic regulated cell death (RCD) that depends on iron and is characterized by the accumulation of lipid peroxides to lethal levels. Ferroptosis involves multiple pathways including redox balance, iron regulation, mitochondrial function, and amino acid, lipid, and glycometabolism. Furthermore, various disease-related signaling pathways also play a role in regulating the process of iron oxidation. In recent years, with the emergence of the concept of ferroptosis and the in-depth study of its mechanisms, ferroptosis is closely associated with various biological conditions related to kidney diseases, including kidney organ development, aging, immunity, and cancer. This article reviews the development of the concept of ferroptosis, the mechanisms of ferroptosis (including GSH-GPX4, FSP1-CoQ1, DHODH-CoQ10, GCH1-BH4, and MBOAT1/2 pathways), and the latest research progress on its involvement in kidney diseases. It summarizes research on ferroptosis in kidney diseases within the frameworks of metabolism, reactive oxygen biology, and iron biology. The article introduces key regulatory factors and mechanisms of ferroptosis in kidney diseases, as well as important concepts and major open questions in ferroptosis and related natural compounds. It is hoped that in future research, further breakthroughs can be made in understanding the regulation mechanism of ferroptosis and utilizing ferroptosis to promote treatments for kidney diseases, such as acute kidney injury(AKI), chronic kidney disease (CKD), diabetic nephropathy(DN), and renal cell carcinoma. This paves the way for a new approach to research, prevent, and treat clinical kidney diseases.

## 1 Introduction

Cell death is a finely regulated process that occurs through different molecular pathways. Apoptosis is an active, programmed mode of cell death, while necrosis is classically defined as uncontrolled, accidental cell death (1). In recent years, various forms of cell death, including programmed necrosis, ferroptosis, pyroptosis, and mitochondria permeability transition-regulated necrosis, have gained attention (2, 3). The term “ferroptosis” was introduced by Dixon et al. in 2012 to describe a cell death form induced by the small molecule erastin (4). It inhibits cysteine import, leading to glutathione (GSH) depletion and glutathione peroxidase 4 (GPX4) lipid peroxidase inactivation. Lipid peroxidation is a downstream feature of ferroptosis, where the accumulation of lipid peroxidation products and reactive oxygen species (ROS) generated by iron metabolism lead to membrane integrity loss through unknown mechanisms (5). Ferroptosis exhibits distinct cellular morphology and function compared to necrosis, apoptosis, and autophagy. It does not display the typical morphological features of necrosis, such as swollen cytoplasm and organelle or cell membrane rupture, nor does it show the characteristic features of traditional apoptosis, such as cell shrinkage, chromatin condensation, and formation of apoptotic bodies (6, 7). Morphologically, ferroptosis is mainly characterized by mitochondrial shrinkage, increased membrane density, and reduction or disappearance of mitochondrial cristae, differentiating it from other cell death modes (8).

In recent years, there has been an increasing recognition of the importance of non-apoptotic cell death mechanisms in elucidating the molecular processes that regulate cell death. However, under normal physiological conditions, these alternative mechanisms of regulating cell death largely remain unknown (8). In this review, we focus on ferroptosis, a form of regulated cell death dependent on iron and involving lethal, iron-catalyzed lipid damage. Ferroptosis is significantly controlled by lipid repair systems including GSH and GPX4, as well as pharmacological interventions, and relies on various pro-survival enzyme reactions (9). Therefore, ferroptosis relies on the interplay of iron, sulfhydryl, and lipid metabolism pathways, with renal tissues particularly susceptible to oxidative-reductive imbalances (10, 11). Recent studies have confirmed the significant role of ferroptosis in the pathophysiology of various kidney diseases, emerging as a new focus of research in the field of renal fibrosis (12). Renal tubules are essential components of the kidney and are vulnerable to damage from factors such as hypoxia, toxins, metabolic disorders, and aging. In response to injury, renal tubular epithelial cells undergo morphological changes and secrete bioactive molecules, driving interstitial inflammation and fibrosis, ultimately leading to the development of chronic kidney disease (CKD) and end-stage renal disease (ESRD) (13, 14). Although specific targeted therapy for renal fibrosis is currently lacking, recent studies suggest that inhibiting ferroptosis may alleviate renal fibrosis. Researchers are investigating the mechanisms by which ferroptosis regulates renal fibrosis in the hope of developing new treatment strategies to delay disease progression, reduce the incidence of ESRD, and lower mortality rates (15–17). Additionally, there is increasing evidence indicating that ferroptosis plays a crucial role in the development of various other kidney diseases, including acute kidney injury (AKI), diabetic nephropathy (DN), renal cell carcinoma, polycystic kidney disease, among others (18–21).

However, there are still gaps in our understanding of the triggering, execution, and propagation mechanisms of ferroptosis in kidney diseases that require further research. This article summarizes current research on ferroptosis, its potential mechanisms, and its role in the progression of various kidney diseases, aiming to provide insights and information for the prevention and treatment of these devastating diseases (**Table 1**).

**Table 1:** Comparison of characteristics of different types of programmed cell death.

Types of cell death	Ferroptosis	Apoptosis	Necroptosis,	Pyroptosis,	Autophagy
Biological characteristics	Inhibition of system X_c_^-^ and GPX4, reduced cysteine uptake, depletion of GSH, iron accumulation, lipid peroxidation	Nucleosomal DNA fragmentation, Caspase activation, mitochondrial membrane potential decrease	Decreased ATP levels, activation of RIP1 and RIP3	Activation of caspase-1-dependent inflammasome and release of pro-inflammatory cytokines	Formation and degradation of autophagosomes,
Morphological features	Cell swelling, increased mitochondrial membrane density, outer membrane rupture, reduced or lost mitochondrial cristae	Membrane blebbing, cell shrinkage, nuclear fragmentation	Plasma membrane rupture, cell swelling, organelle disarray, chromatin condensation	Plasma membrane rupture, organelle swelling, nuclear condensation	Formation of double-membrane lysosomes
Regulatory genes	ATP5G3, GPX4, Nrf2, RPL8,RAS,SLC7A11	Bcl-2, Bax, Bak, Caspase, P53, Fas	RIPK1,RIPK3, MLKL	Caspase-1, Gasdermins, NLRP3	mTOR, LC3,ATG5,Beclin 1,ULK1,AMPK
Inducers	Erastin, Glutamate, SAS, SRS, Sorafenib, Artemisinin	Apoptosis protein A, Hypoxia, Fasl, Staurosporine, UNC5B	TNFα, Fasl, TWEAK	ZnO-NPs, Ivermectin	Rapamycin, Simvastatin, Valproic acid, Thapsigargin
Inhibitors	Ferrostatin-1, Liproxstatin-1, Deferoxamine, VitE, U0126, DFO, CHX	NAIP, CTX1,c-IAP1/2, XIAP, ILP-2, Survivin, Z-VADFMK	Necrostatin-1, NSA, Kongensin-A	Necrosulfonamide	BafilomycinaA1,3-MA, LY294002,wortmannin, Spautin-1
Inflammatory features	Pro-inflammatory	anti-inflammatory	Pro-inflammatory	Pro-inflammatory	anti-inflammatory

## 2 Overview of ferroptosis

The discovery process regarding ferroptosis began in 2001 when TAN et al. observed that exogenous glutamate inhibits cystine uptake through the cystine/glutamate antiporter system, depleting GSH and inducing an increase in ROS levels and intracellular Ca2+ influx, causing a form of programmed cell death in neuronal cells distinct from apoptosis (22). In 2003, Dolam et al. identified a compound, Erastin, through screening compounds with genotype-selective properties, that exhibited selective lethal effects on cells with RAS gene mutations. Erastin induces a non-apoptotic cell death pathway in RAS gene mutant cells (23). Subsequently, in 2008, Yang et al. identified two new compounds, Ras-selective lethal compound (RSL)3 and RSL5, which induced a non-apoptotic form of cell death similar to Erastin (24). In 2013, Dixon et al. discovered that Erastin triggered an iron-dependent, non-apoptotic form of cell death, and officially named this cell death mechanism “ferroptosis”. Ferroptosis differs from apoptosis, necrosis, and autophagy: morphologically, there are no chromatin condensation and marginalization as in apoptosis, cytoplasmic and organelle swelling as in necrosis, and double-membrane-wrapped vesicles as in autophagy. A unique morphological feature of ferroptosis is that mitochondria appear smaller than normal, with increased membrane density (25). Further research has shown that Erastin and similar ferroptosis inducers induce cell death by reducing the synthesis of GSH, leading to the inactivation of GPX4. This results in increased lipid peroxidation, ultimately causing cell death. On the other hand, ferroptosis inducers such as RSL3 do not affect the concentration of GSH in cells, instead, they directly bind to GPX4, leading to increased lipid peroxidation and subsequent cell death (26). Cysteine is the rate-limiting substrate in the biosynthesis of reduced GSH in a biological system. It is either taken up by cells in its oxidized form (cystine) through the cysteine/glutamate antiporter (Xc- system) and the sodium-dependent neutral amino acid transporter B(0)AT1 (SLC6A19), or in its reduced form, cysteine, entering cells through neutral amino acid transporters, or being produced through transsulfuration from endogenous sources (27, 28). GSH is the most abundant reducing agent in mammalian cells, playing a crucial role in iron-sulfur cluster biogenesis. It also serves as a cofactor for various enzymes including glutathione peroxidase (GPX) and glutathione S-transferase (29). Genetically, it has been demonstrated that the GSH synthesis, the Xc- system, and GPX4 all contribute to protecting cells from death under various oxidative stress conditions, especially under conditions leading to sulfhydryl depletion, including the inhibition of Xc- system activity (30). With the establishment of the roles of GSH synthesis, the Xc- system, and GPX4 in ferroptosis, we can now contextualize all these early studies within the framework of iron-dependent cell death.

The concept of ferroptosis was first proposed by Dixon et al (31) in 2012 as an iron-dependent form of cell death characterized by intracellular ROS accumulation, distinct from apoptosis. Before the introduction of the term “ferroptosis”, relevant inducers had already been discovered. In 2003, Dolma et al. first discovered a new compound, erastin, capable of killing tumor cells with mutant RAS oncogenes (23, 32). The cell death did not involve changes in the nucleus or activation of caspase-3. Subsequently, Yang et al. discovered another compound, the RSL3, which can induce this form of cell death. The cell death caused by these two compounds was later confirmed to be ferroptosis (24, 33).

**Figure 1:**
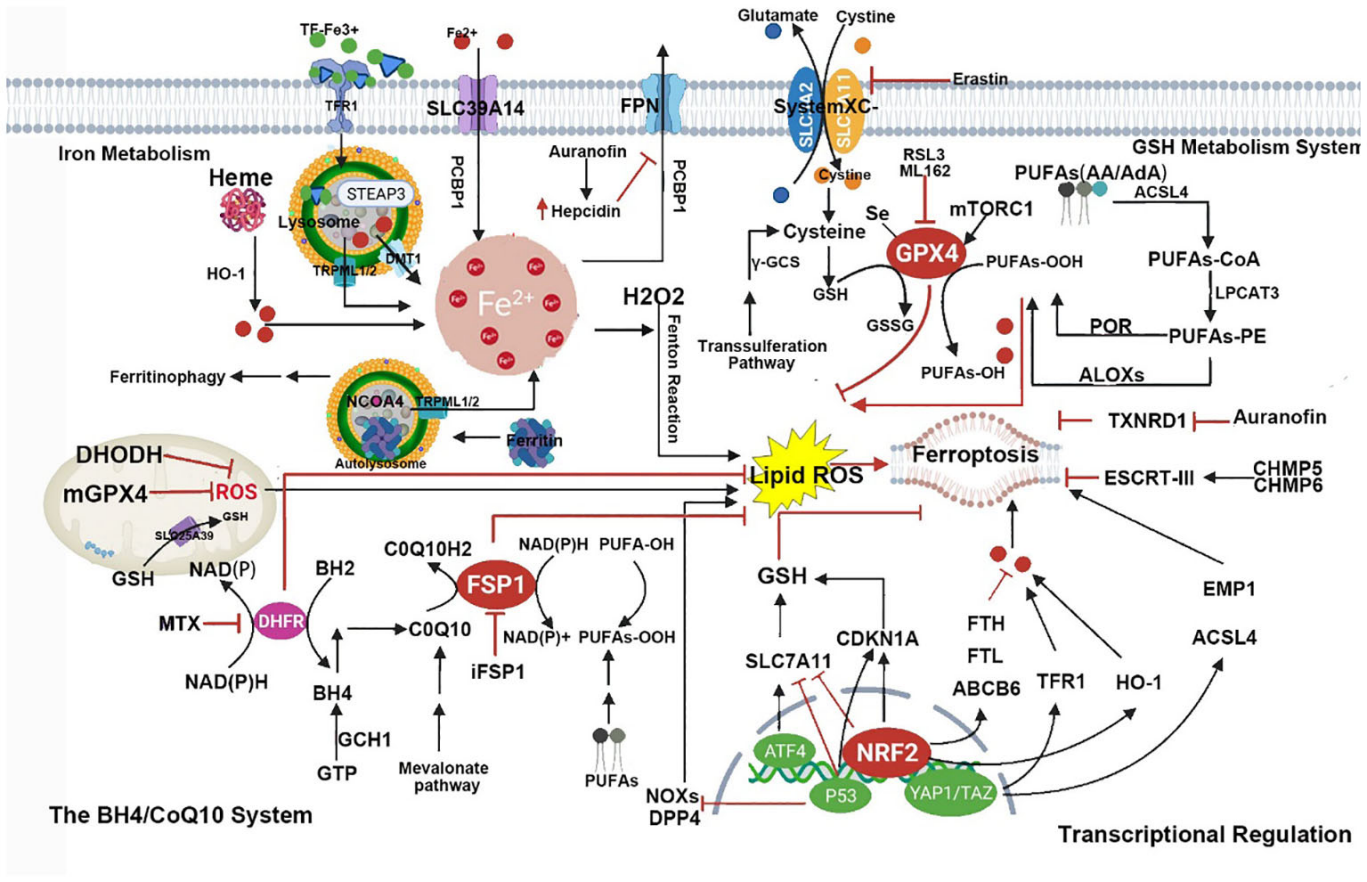
This figure shows the metabolic pathways involved in iron-dependent cell death. Iron-dependent lipid peroxidation drives iron death at the cellular level. Several aspects of iron metabolism, such as absorption, storage, and utilization, play important roles in regulating iron death. Additionally, activation of long-chain fatty acid CoA ligase 4 (LACS4), lysophosphatidyltransferase 5 (LPLAT5), lipid oxidase (LOX), or NADPH oxidase (NOX) in the lipid metabolism pathway promotes lipid peroxidation and iron death. The classic iron death suppression pathway involves the cysteine-glutamate reverse transporter (Xc-system), which induces the biosynthesis of GSH by facilitating cysteine (Cys) uptake. Using GSH as a cofactor, GPX4 reduces phospholipid hydroperoxides to their respective alcohols. The peroxidation of phospholipids can also be suppressed by the iron death inhibitor factor 1 (FSP1)-coenzyme Q10 (CoQ10) system. Furthermore, iron death is regulated by iron metabolism, including absorption, transport, storage, and utilization of iron. At the cellular level, non-heme iron enters cells through transferrin receptor 1 (TFR1)-mediated iron uptake by transferrin (TF) binding, or iron uptake independent of TF mediated by solute carrier family 39 member 14 (SLC39A14, also known as zinc transporter ZIP14). Additionally, iron engulfment mediated by heme degradation and nuclear receptor coactivator 4 (NCOA4) increases the labile iron pool (LIP), making cells more sensitive to iron death via the Fenton reaction. FPN, ferritin, Glu represents glutamate; GSSG, oxidized glutathione; HO1, heme oxygenase 1; KEAP1, kelch-like ECH-associated protein 1; NRF2, nuclear factor E2-related factor 2; PUFA, polyunsaturated fatty acid; PUFA-CoA, polyunsaturated fatty acid-coenzyme A; PUFA-PL, phospholipid containing polyunsaturated fatty acids (PUFA); and STEAP3, metalloreductase STEAP3.

As a form of RCD, ferroptosis differs from apoptosis, necrosis, autophagy, and other forms of cell death. Morphologically, cells undergoing ferroptosis exhibit significant mitochondrial contraction, increased mitochondrial membrane density, loss of mitochondrial cristae, and rupture of the mitochondrial outer membrane (8); Biochemically, inhibiting the cystine/glutamate antiporter system, known as system Xc on the cell membrane leads to a decrease in the activity of GPX4. This inhibition directly or indirectly results in intracellular depletion of GSH. Consequently, GPX4 is unable to utilize GSH to convert lipid hydroperoxides into lipid alcohols, leading to an inability to effectively clear ROS and lipid reactive species generated by lipid membrane damage, disruption of the mitochondrial electron transport chain, and potentially by iron release from iron-containing enzymes. This cascade eventually induces cell death, known as ferroptosis (8, 34). A pathway map of the current mechanism of ferroptosis is summarized in **Figure 1**.

### 2.1 The mechanism of ferroptosis

#### 2.1.1 The GPX4/GSH signaling pathway in ferroptosis

GSH is the primary antioxidant involved in intracellular antioxidant stress, participating in numerous essential cellular metabolic activities such as the removal of ROS, DNA and protein synthesis, and signal transduction. Severe oxidative stress can cause damage to cellular lipids, proteins, DNA, and even lead to cell death. Oxidative stress can be induced in two main ways: directly increasing ROS levels or impairing antioxidant defense systems. Among the members of the GPX family, GPX4 acts as an inhibitory protein in lipid peroxidation. It reduces lipid hydroperoxides to lipid alcohols, preventing ROS accumulation and playing a role in inhibiting cell ferroptosis (35). GPX4’s activity depends on system xc, a widely distributed amino acid antiporter in the phospholipid bilayer consisting of a light chain subunit (solute carrier family 7A11, *SLC7A11*) and a heavy chain subunit (solute carrier family 3 member 2, *SLC3A2*). System xc can mediate the exchange of extracellular cystine and intracellular glutamate across the cell membrane. Cysteine, derived from cystine, is a rate-limiting substrate for the synthesis of the antioxidant GSH (36). Therefore, the import of cystine through this transporter is crucial for GSH production and oxidative protection. Ferroptosis is primarily caused by an imbalance between the generation and degradation of intracellular lipid ROS within cells.

Studies have shown that inhibiting system X_c_^-^ using erastin and sulfasalazine in cancer cells cultivated in covered dishes results in a unique form of iron-dependent cell death known as ferroptosis (31). The small molecule erastin inhibits system X_c_^-^, impeding GSH absorption. GSH is a necessary cofactor for *GPX4* activity. Consequently, GPX4 activity decreases, reducing cellular antioxidant capacity, leading to lipid peroxidation accumulation and inducing oxidative cell death, known as ferroptosis. (1S,3R)-RSL3, also known as *RSL3*, and *ML162* (also known as DPI7) can deactivate GPX4, triggering ferroptosis in cells (33).

#### 2.1.2 Ferroptosis and polyunsaturated fatty acids

**Figure 2:**
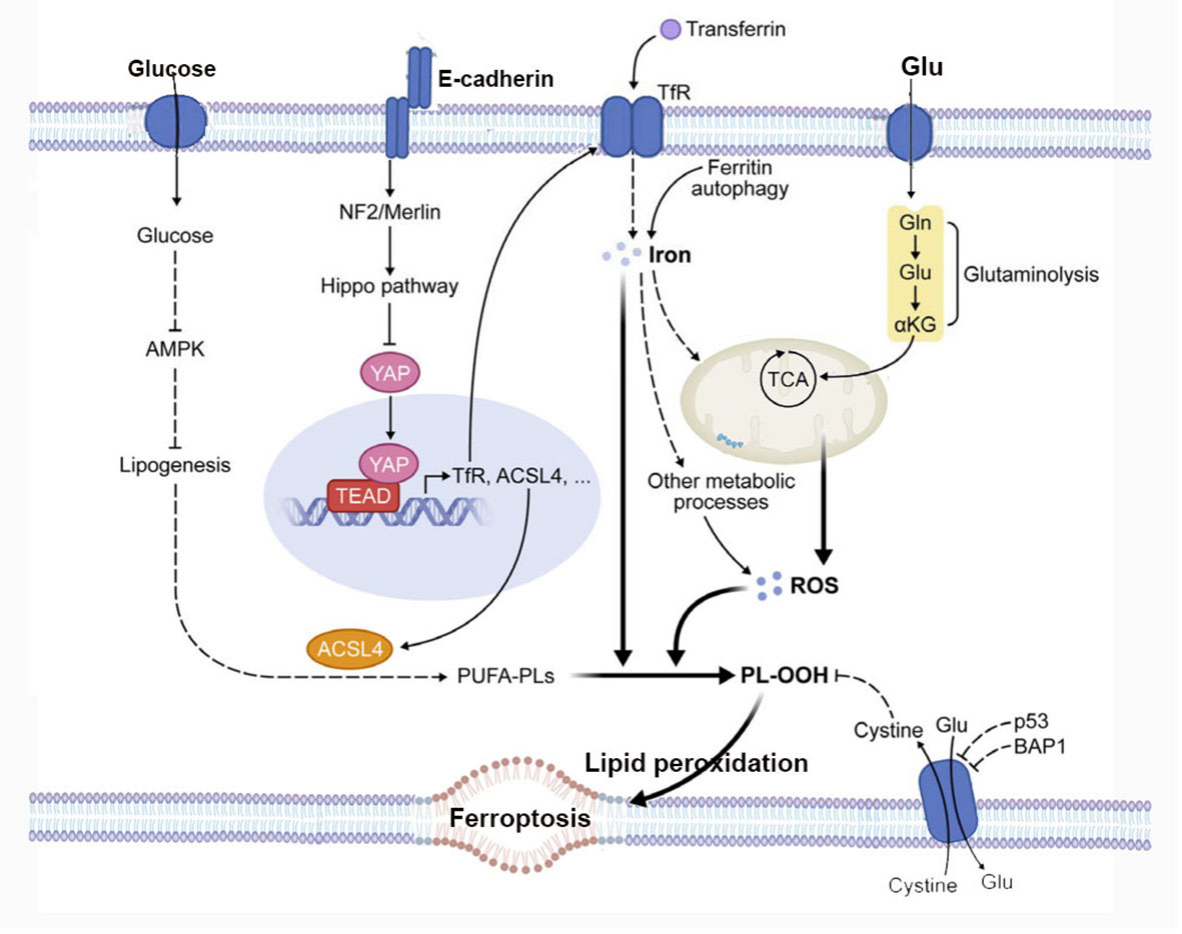
The figure depicts the regulation of ferroptosis by multiple metabolic events (such as lipogenesis, autophagy, and mitochondrial TCA cycle) and signaling pathways (such as E-cadherin-NF2-Hippo-YAP pathway, glucose-regulated AMPK signaling, and p53 and BAP1 tumor suppressor function). See text for details. TfR, transferrin receptor; PL-OOH, phospholipid hydroperoxide; PUFA-PL, phospholipid with polyunsaturated fatty acid chain; ROS, reactive oxygen species; TCA, mitochondrial TCA cycle; Gln, glutamine; Glu, glutamate; αKG, α-ketoglutarate.

Ferroptosis is characterized by lipid peroxidation, a process regulated by the system xc−/GPX4/GSH signaling pathway. Another condition for cells to undergo ferroptosis is the presence of polyunsaturated fatty acids (PUFAs), including arachidonic acid (AA) and docosapentaenoic acid. PUFAs contain easily extractable bis-allylic hydrogen atoms, making them prone to lipid peroxidation, which is necessary for executing ferroptosis (37). Therefore, the abundance and localization of PUFAs determine the extent of lipid peroxidation occurring in cells, thereby influencing the degree of ferroptosis’s action. Free PUFAs serve as substrates for the synthesis of lipid signaling mediators, but they must be esterified into membrane phospholipids and undergo oxidation to become signals for ferroptosis. In cells undergoing ferroptosis, the AA is significantly depleted, and lipid fragments derived from AA are detected in the conditioned media of GPX4^-/-^ mouse embryonic fibroblast cultures (34). Long-chain acyl CoA synthetase 4 (ACSL4) and lysophosphatidylcholine acyltransferase 3 (LPCAT3) encode enzymes involved in incorporating AA into membrane phospholipids. The absence of ACSL4 and LPCAT3 can prevent ferroptosis induced by GPX4 inhibitors RSL3 and ML162 (38). This indicates that the execution of cell ferroptosis in the presence of highly oxidative PUFAs such as AA can only occur after the direct or indirect (i.e., induced by GSH depletion) inactivation of GPX4 (35). Existing research suggests that this could be another potential point of regulation for ferroptosis, by modulating the enzymes involved in the biosynthesis of membrane phospholipids containing PUFAs to trigger or block ferroptosis (39, 40). The summary of metabolism and cell signaling in ferroptosis is shown in **Figure 2**.

#### 2.1.3 Iron metabolism and ferroptosis

Iron is an essential trace element in the human body, playing multiple important biological roles, including inducing ATP production, participating in DNA and hemoglobin synthesis, and many other physiological activities (41). Due to its ability to accept and donate electrons, the accumulation of ferrous ions can lead to oxidative damage and even cell death (42). In mammalian cells, the absorption pathways for non-heme and heme iron involve various transport proteins or receptors, providing iron for subsequent lipid peroxidation processes. Elevated intracellular iron levels, particularly high ferrous ions levels, can lead to lipid peroxidation (43). Cellular iron homeostasis is closely related to the absorption, storage, circulation, and utilization of iron (44). In general, extracellular Fe3+ ions first bind to transferrin (TFR), and then enter the cell through the transferrin receptor 1 (TFR1) for storage in the form of the ferritin complex (primarily ferritin) (45).

Fe^3+^ ions are reduced to Fe^2+^ ions and then transported and stored in the cellular iron pool, while excess Fe^2+^ ions are stored in ferritin (46). Ferritin is a complex of iron storage proteins consisting of ferritin light chain and ferritin heavy chain 1 (FTH1). In cases of disrupted iron metabolism, low expression of FTH1 and overexpression of TFR1 often lead to excessive accumulation of Fe^2+^ ions, inducing the production and accumulation of large amounts of ROS through the Fenton reaction, ultimately promoting cell ferroptosis (47). With increased iron supplementation, tissue iron concentrations rise, potentially exceeding the body’s binding capacity, leading to the formation of non-transferrin-bound iron (NTBI). Organs such as the liver and kidneys are sensitive to iron, and their absorption and clearance of iron differ from the reticuloendothelial system, possibly causing tissue iron deposition and iron overload (48). During the iron cycling process, NTBI and certain unstable ferrous species are prone to oxidation and reduction through Fenton and Haber-Weiss reactions, generating hydroxyl radicals (·OH). These radicals can damage large molecules such as lipids, proteins, and nucleic acids, causing oxidative stress (49). The products of lipid peroxidation chain reactions exhibit high biological activity (50). It can damage DNA, proteins, and enzyme activity, serving as molecular signals activating pathways that lead to cell death (51). Lipid peroxidation plays a driving role in ferroptosis and can be accomplished through non-enzymatic or enzymatic reactions. Compared to saturated fatty acids and monounsaturated fatty acids, PUFAs are more prone to lipid peroxidation and ferroptosis (52). The formation of PUFA coenzyme A derivatives is a necessary condition for initiating ferroptosis. Enzymes involved in regulating PUFA biosynthesis in membrane phospholipids can either trigger or prevent ferroptosis (52). Furthermore, studies have found that altering the intracellular iron content can change the sensitivity of cells to ferroptosis. Increasing transferrin and transferrin receptor-1 can boost cellular iron levels, thereby promoting ferroptosis (53).

**Figure 3:**
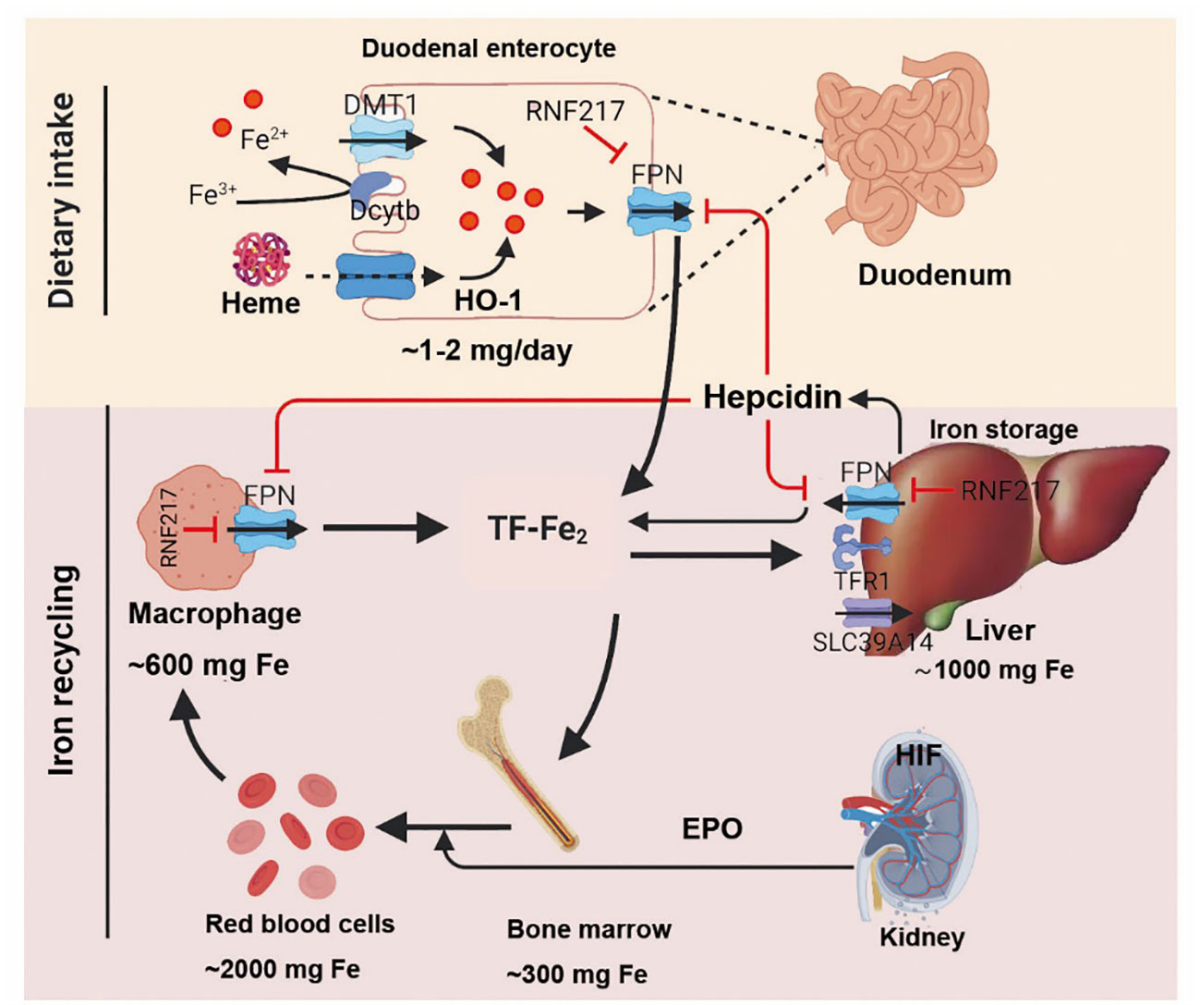
After intake of iron, Fe3+ is reduced by dcytb and then transported into enterocyte through DMT1. Dietary heme is absorbed by unknown mechanism and degraded in enterocyte by HO-1. Once exported by FPN, Fe3+ binds to transferrin (diferric transferrin, TF-Fe2), travels to tissues, and largely utilized in new red blood cells. Macrophage degraded senescent RBCs to recycle iron. Once needed, EPO, released by kidney, promotes erythropoiesis by HIF signaling pathway. The iron utilization of erythroid marrow and its recycling by macrophages represent the major iron circulation. Excess iron can be stored in hepatocytes through TFR1-mediated TF-Fe2 or SLC39A14-participated non-transferrin-bound iron (NTBI). The release of iron from enterocyte, red blood cells, and macrophages is precisely controlled by FPN, the body’s sole iron exporter, to maintain a relatively stable iron level. The peptide hepcidin, the master regulator of systemic iron homeostasis, is a circulating hormone synthesized by the liver. Recently, we identified RNF217 as a novel E3 ligase for mediating FPN degradation. Dcytb, duodenal cytochrome b; DMT1, divalent metal transporter 1; EPO, erythropoietin; FPN, ferroportin; TFR1, transferrin receptor 1; HO-1, heme oxygenase 1; HIF, hypoxia induced factor; RBCs, red blood cells; NTBI, non-transferrin-bound iron.

**Figure 4:**
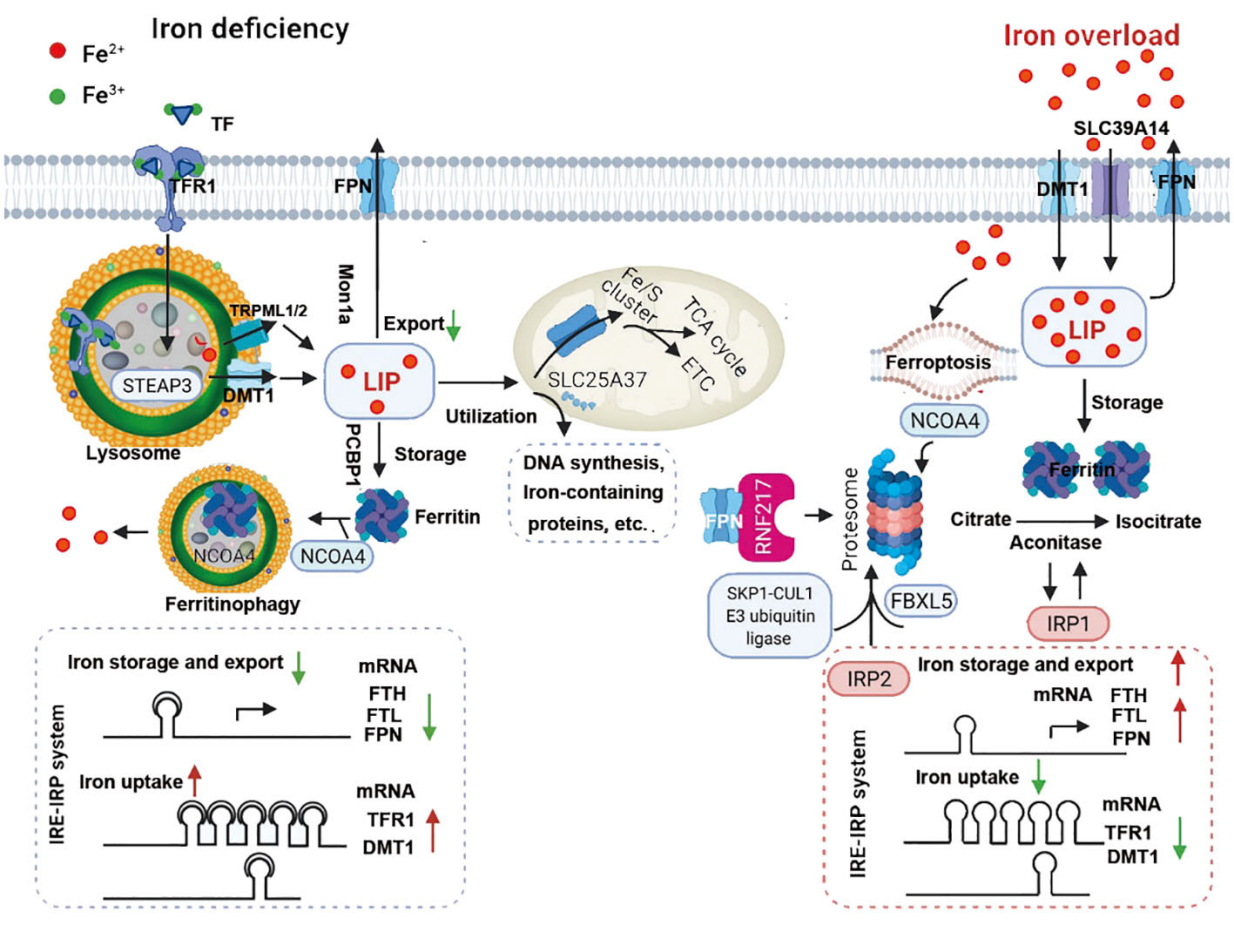
Under iron-deficient conditions (left), the majority of iron is bound to transferrin (TF), which binds to the transferrin receptor 1 (TFR1) at the cell surface followed by receptor-mediated endocytosis, resulting in ferric iron being released from TF and reduction to ferrous iron by an lysosomal reductase such as STEAP3. The ferrous iron is then transported into the lysosomal membrane by DMT1 and TRPML1/2, where it becomes part of the labile iron pool in the cytosol. Labile iron can be stored in the iron-storage protein ferritin or used to synthesize heme and iron-sulfur clusters in the mitochondria or in the cytosol. Iron can also be exported from the cell by the body’s sole iron exporter, ferroportin (FPN). In addition, the IRE/IRP system regulates the expression of iron-related proteins such as TFR1, ferritin and FPN, upregulating TFR1 and DMT1 expression and downregulating FPN and FTH/FTL expression. During iron overload (right), hepcidin expression is upregulated by either the canonical bone morphogenetic protein (BMP)/SMAD pathway or by IL-6-pSTAT3 inflammatory signaling, which in turn limits iron absorption by increasing FPN degradation. In response to excess iron, BMP6, together with HJV, activates type 1 (Alk2/3)and type 2 (BMPR2, ACVR2A) BMP serine threonine kinase receptors to phosphorylate R-SMAD (receptor-activated SMAD), leading to activation of BMP/SMAD signaling pathway. High concentration of TF-Fe2 interact with TFR1, resulting in forming complex of TFR2/HJV/HFE to enhance the BMP/SMAD signaling in regulating hepcidin. TMPRSS6 inhibits BMP/SMAD signaling by cleaving HJV. The IRP system not only downregulates iron uptake-related genes such as TFR1 and DMT1 expression, it also upregulates FPN and FTH/FTL expression. IRP2 mediated by SKP1-CUL1 E3 ubiquitin ligase and NCOA4 are degraded, while IPR1 works as aconitase to convert citrate to isocitrate due to conformational change. RNF217 is a recently identified E3 ligase that regulates the degradation of FPN. ACVR2A, activin receptor type-2A; ALK, activin receptor-like kinase; BMP6, bone morphogenetic protein 6; BMPR2, bone morphogenetic protein receptor type 2; DMT1, divalent metal transporter 1; EPO, erythropoietin; ERFE, erythroferrone; ETC, electron transport chain; FBXL5, F-box/LRR-repeat protein 5; FPN, ferroportin; FTH, ferritin heavy chain; FTL, ferritin light chain; JAK, Janus kinase; LIP, labile iron pool; NCOA4, nuclear receptor coactivator 4; NTBI, non-transferrin-bound iron; HJV, hemojuvelin; IL-6, interleukin 6; IRE, iron-responsive elements; IRP, iron-regulatory proteins; SLC39A14, solute carrier family 39 member 14; SMAD4, SMAD family member 4; SMAD7, SMAD family member 7; STAT3, signal transducer and activator of transcription 3; STEAP3, six-transmembrane epithelial antigen of prostate 3; TCA cycle, tricarboxylic acid cycle; TFR1, transferrin receptor 1; TFR2, transferrin receptor 2; TMPRSS6, transmembrane protease serine 6; TRPML1/2, Mucolipin TRP channel 1/2;UTRs, untranslated regions.

TFRC is the gene encoding the transferrin receptor. The transferrin receptor is essential for cellular uptake of transferrin-iron complexes. Silencing the *TFRC* encoding gene can effectively prevent ferroptosis induced by erastin or cysteine deficiency. Changes in the transcription of iron-regulatory genes such as *IREB2*, *FBXL5*, *TFRC*, *FTH1*, and *FTL* affect the sensitivity to erastin-induced ferroptosis, with this sensitivity positively correlating with intracellular iron abundance (54). So far, the mechanism of iron ions in ferroptosis remains incompletely understood. While the independent redox action of iron ions cannot be entirely ruled out, the most plausible explanation for chelators preventing ferroptosis is by inhibiting iron ions from donating electrons to oxygen to generate ROS (55). Iron ions are essential for the accumulation of lipid peroxides and the execution of ferroptosis. Therefore, the sensitivity of iron-induced cell death is influenced by iron intake, output, storage, and turnover. The Regulation of systemic iron homeostasis was shown in **Figure 3**. Iron metabolism was shown in **Figure 4**.

#### 2.1.4 Ferroptosis and necroinflammation

Cellular ferroptosis triggers the innate immune system by releasing damage-associated molecules associated with inflammation. Immune cells stimulate inflammatory responses by recognizing the mechanisms of different patterns of cell death mechanisms (56). The ferroptosis inhibitor Ferrostatin-1 can avoid the exacerbation of kidney damage after ferroptosis by blocking the release of necrosis-related alarmone IL-33 and other chemokines and cytokines, thus preventing macrophage infiltration. This indicates the significant relationship between ferroptosis and inflammatory responses (57). When renal tubular epithelial cells undergo ferroptosis or other forms of RCD, their cellular contents are released in the form of DAMPs. These DAMPs bind to different molecular receptors on other cells in the interstitium, leading to the generation of an immune response known as necroinflammation, causing further damage via inflammatory reactions. This process contributes to further inflammation and damage due to the necroinflammatory response (58). Among the numerous damage-associated molecules released by necrotic cells, high-mobility group box 1 protein (HMGB1) receives significant attention. HMGB1 is a non-histone nuclear protein. When tissues or organs are damaged, it is released from damaged cells into the extracellular space or directly as a component of necrotic cell debris. It serves as a danger signal recognized by the immune system, thereby initiating an inflammatory response (59, 60). Current research indicates that HMGB1 can activate the TLR4-MyD88 signaling pathway (61) by binding to Toll-like receptors (TLRs), mainly TLR4. This activation leads to the phosphorylation of p38 MAPK, mediating the activation of mitogen-activated protein kinases (MAPK) and subsequent nuclear transcription. This process promotes the release of more inflammatory factors, thus exhibiting a pro-inflammatory effect. Past research has indicated that HMGB1 is a key regulatory factor in ferroptosis since HMGB1 translocation requires ROS-dependent signaling (62, 63). Ye et al. (64) conducted a study showing that in HL-60/NRASQ61L cells, the ferroptosis inducer erastin increases ROS levels, facilitates cytoplasmic translocation of HMGB1, and promotes cell death. The downregulation of HMGB1 reduces ROS generation induced by erastin and iron-mediated cell death in HL-60/NRASQ61L cells. This suggests that the ferroptosis inducer erastin acts as an activator for HMGB1 cytoplasmic translocation and release and that HMGB1 is a crucial regulatory point in executing ferroptosis.

### 2.2 Other regulatory pathways and significance of ferroptosis

#### 2.2.1 Regulation by System X_c_^-^

X_c_^-^ is a cystine/glutamate antiporter protein that plays a crucial role in regulating cellular redox homeostasis (65). The system X_c_^-^ consists of two subunits: SLC7A11 and SLC3A2, functioning to import cystine into the cell in exchange for glutamate (66). Cystine is then converted to cysteine, and cysteine serves as a precursor to synthesize GSH, an important intracellular antioxidant (67). The interaction mechanism between ferroptosis and the system X_c_^-^ involves the regulation of cellular redox balance. The normal function of the system X_c_^-^ is crucial for maintaining intracellular redox balance. When the system X_c_^-^ is dysfunctional or inhibited, the levels of cysteine and GSH inside the cell decrease, leading to an exacerbation of intracellular oxidative stress, resulting in the accumulation of lipid peroxides and ultimately triggering ferroptosis (68, 69). Therefore, restoring or maintaining the normal function of the system X_c_^-^ can help in maintaining intracellular redox balance, inhibiting the occurrence of ferroptosis, and potentially have therapeutic effects for various diseases, such as cerebral ischemia-reperfusion injury (CIRI) (70, 71).

#### 2.2.2 FSP1/CoQ10/NADPH pathway

Ferroptosis suppressor protein 1(FSP1) is a protein capable of participating in iron-sulfur cluster modifications, transferring iron-sulfur clusters from mitochondria to target proteins (72). Coenzyme Q10 (CoQ10) is an antioxidant that can reduce the generation of free radicals and diminish the occurrence of oxidative stress reactions (73). Nicotinamide adenine dinucleotide phosphate (NADPH) is a reducing coenzyme that provides reducing power and participates in many metabolic pathways (74). Recent research indicates that FSP1 in the FSP1/CoQ10/NADPH pathway can protect mitochondria from oxidative stress damage during ferroptosis processes by regulating the synthesis and transfer of iron-sulfur clusters; CoQ10 reduces the generation of free radicals directly, thereby reducing the occurrence of oxidative stress reactions. NADPH provides reducing power, reducing the severity of oxidative stress reactions, thereby protecting cells from damage caused by oxidative stress reactions (72, 74). In conclusion, the FSP1/CoQ10/NADPH pathway regulates ferroptosis processes through various mechanisms, protecting cells from damage caused by ferroptosis. This discovery provides a new perspective and approach to the treatment of ferroptosis (74).

#### 2.2.3 DHODH pathway

**Figure 5:**
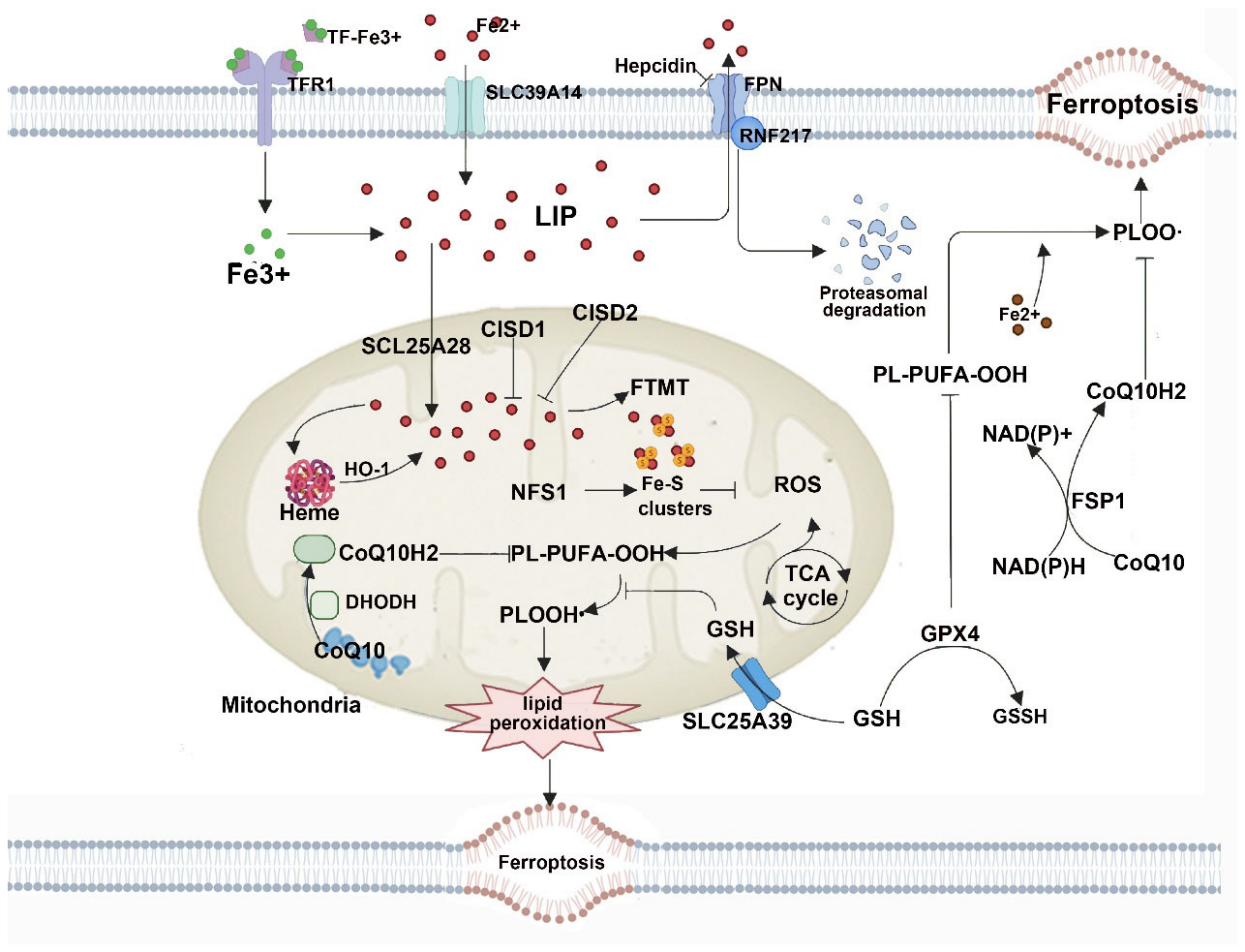
Mitochondria host a wide range of key metabolic processes (such as the tricarboxylic acid (TCA) cycle) and are a major source of reactive oxygen species (ROS). Separate mitochondria-localized defense systems have evolved to prevent mitochondrial lipid peroxidation and ferroptosis. For example, either the mitochondrial version of phospholipid hydroperoxide glutathione peroxidase 4 (GPX4) or dihydroorotate dehydrogenase (quinone), mitochondrial (DHODH) can specifically detoxify mitochondrial lipid peroxides. Moreover, the mitochondria-specific form of ferritin (FTMT) protects mitochondria from iron overload-induced oxidative injury, and mitoNEET (also known as CISD1) suppresses ferroptosis by limiting mitochondrial iron uptake. CoQ10, coenzyme Q10; FPN, ferroportin; FSP1, ferroptosis suppressor protein 1; GSH, glutathione; GSSG, glutathione disulfide; HO1, haem oxygenase 1; LIP, labile iron pool; PL-PUFA-OOH, polyunsaturated fatty acid-containing phospholipid hydroperoxides; PLOO·, phospholipid peroxyl radical; RNF217, E3 ubiquitin protein ligase RNF217; SLC25A39, probable mitochondrial glutathione transporter SLC25A39; SLC39A14, solute carrier family 39 member 14; TF, transferrin; TFR1, transferrin receptor protein 1.

Dihydroorotate dehydrogenase (DHODH) is a mitochondrial inner membrane enzyme that plays a role in important metabolic pathways such as cytochrome P450, purine synthesis, and fatty acid metabolism (75, 76). Research indicates that the DHODH pathway can protect cells from oxidative stress and mitochondrial damage through various mechanisms, including downregulating ROS levels, maintaining mitochondrial membrane potential, and inhibiting cell apoptosis (77, 78). DHODH inhibits ROS production and increases mitochondrial membrane potential, thus preventing cells from undergoing mitochondrial permeability transition and apoptosis, safeguarding cells from oxidative stress and mitochondrial damage. This provides a novel approach and pathway to shield cells from ferroptosis (79, 80). The regulatory role of mitochondria in ferroptosis shown in **Figure 5**.

#### 2.2.4 GTP cyclohydrolase 1 (GCH1)/tetrahydrobiopterin (BH4)

The GCH1/BH4 pathway is an intracellular enzymatic pathway involved in various crucial cellular metabolic processes (81, 82). GCH1 is the rate-limiting enzyme for BH4, and the GCH1/BH4 pathway can regulate redox reactions and cell apoptosis, thereby protecting cells from the impact of oxidative stress and mitochondrial damage (83, 84). Recent research indicates that the GCH1/BH4 pathway also plays a critical role in ferroptosis, where GCH1 increases BH4 synthesis, decreases ROS levels, mitigates oxidative stress, and promotes cell survival. Furthermore, BH4 can regulate multiple cellular signaling pathways and metabolic pathways, further reducing oxidative stress and cell death, thereby protecting cells from the damage caused by ferroptosis (82, 83, 85).

#### 2.2.5 Others

Currently, there are multiple endogenous defense pathways in cells to counteract ferroptosis, including *GSH-GPX4*, *FSP1-CoQ1*, *DHODH-CoQ10*, *GCH1-BH4*, as well as *MBOAT1/2* (86, 87). Lang et al. revealed through whole-genome CRISPR activation screening that MBOAT1 and MBOAT2 are novel inhibitors of ferroptosis (88). They suppress ferroptosis by reshaping phospholipids, a mechanism independent of *GPX4* or *FSP1* (88). The research also uncovered that transcription of *MBOAT1* and *MBOAT2* is upregulated by estrogen receptor (ER) and androgen receptor (AR), respectively. Inducing ferroptosis in combination with ER or AR antagonists significantly inhibits the growth of ER-positive breast cancer or AR-positive prostate cancer, offering a novel therapeutic approach for cancers with specific genetic backgrounds. Additionally, Interleukin 4 Induced Protein 1 (IL4i1) is an extracellular matrix enzyme that metabolizes tryptophan and its metabolites to regulate the intracellular redox balance (89, 90). Recent studies indicate that IL4i1 plays a crucial role in ferroptosis by modulating cellular redox balance through the metabolism of tryptophan and its derivatives. This helps in reducing ROS levels, mitigating oxidative stress, and protecting cells from ferroptosis-induced damage (91). The relationship between ferroptosis and IL4i1 is intricate, necessitating further in-depth exploration of their interactions and regulatory mechanisms to offer new insights and avenues for ferroptosis treatment.

### 2.3 The relationship between ferroptosis and other programmed cell death responses

Ferroptosis is intricately linked to biological processes, including autophagy, endoplasmic reticulum stress, and inflammation, among others, and may be involved in the reciprocal regulation of recalcitrant diseases. Therefore, a deeper exploration of the relationship and regulatory effects between these processes can offer a solid foundation for disease treatment.

#### 2.3.1 The relationship between ferroptosis and autophagy

Appropriate autophagy has evolved into a pro-survival response for cells, but excessive autophagy, especially selective autophagy, and impaired lysosomal activity may promote cellular ferroptosis (92). The degradation of ferritin can be completed through ferritinophagy, a selective autophagy process mediated by Nuclear Receptor Co-activator 4 (NCOA4). Knocking out NCOA4 can inhibit ferritin degradation, preventing ferroptosis caused by free iron in fibroblasts and pancreatic cancer cells, thereby directly linking autophagy to ferroptosis (92). The autophagy-dependent lysosomal degradation of ferritin also enhances artemisinin-induced ferroptosis in cancer cells, which is another mechanism during ferroptosis that leads to ferritin degradation (93). Clockophagy is a recently discovered form of selective autophagy. *ARNTL* is a circadian rhythm transcription factor that inhibits the transcription of Egln2 and activates the survival transcription factor HIF1A, thereby inhibiting ferroptosis. Targeting this novel ARNTL-EGLN1-HIF1A pathway may enhance the anticancer activity of ferroptosis inducers (94).

#### 2.3.2 Ferroptosis and endoplasmic reticulum stress

Dixon et al. found that Erastin induces endoplasmic reticulum stress by activating the PERK-eIF2α-ATF4-CHOP pathway and upregulating the expression of the apoptotic protein PUMA, indicating that endoplasmic reticulum stress may be involved in ferroptosis (95). Furthermore, HSPA5 is a molecular chaperone associated with endoplasmic reticulum stress that can bind to GPX4 to inhibit protein kinase-induced degradation of GPX4, thereby suppressing ferroptosis in pancreatic cancer cells. Chen et al. (96) identified elevated levels of ATF4 in human glioblastoma, and pharmacological or genetic inhibition of System X_c_^-^ can attenuate ATF4-induced cancer cell proliferation. Additionally, ATF4 promotes tumor-mediated neurotoxicity and tumor angiogenesis, which can be alleviated by ferroptosis inducers such as Erastin and RSL3. Therefore, inhibition of ATF4 may be an effective target for reducing tumor growth by sensitizing cancer cells to ferroptotic cell death.

#### 2.3.3 Ferroptosisis and inflammation

Unlike immunologically silent apoptosis, ferroptosis is immunogenic, as cells undergoing ferroptosis release cell contents including DAMPs and alarm proteins due to plasma membrane rupture, amplifying cell death and triggering a cascade of inflammation-related responses (97). In a mouse model of crystal-induced acute kidney injury (AKI), inhibitors of ferroptosis suppressed the expression of pro-inflammatory cytokines and the infiltration of neutrophils into the damaged tissue (98). Kang et al. (99) demonstrated that Gpx4 expressed in myeloid cells plays a crucial role in lipid peroxidation, inflammasome activation, and release of DAMPs in the setting of sepsis, with Gpx4 deletion leading to increased lethality in sepsis conditions. Qi et al. (100) found that in a mouse model of nonalcoholic steatohepatitis (NASH) induced by methionine/choline deficiency (MCD) feeding, levels of inflammatory cytokines including TNF-α, IL-1β, and IL-6 protein increased significantly after treatment with the ferroptosis inducer RSL3. However, mice fed with an MCD diet and treated with sodium selenite (a GPX4 activator) showed elevated hepatic GPX4 levels, reduced lipid peroxidation, and decreased severity of NASH.

## 3 Ferroptosis inducers and inhibitors

Ferroptosis inducers and inhibitors act by modulating key mechanisms in the ferroptosis pathway mentioned above to respectively promote or inhibit iron-dependent cell death (24, 74, 98, 101–108). Inducers mainly include (1) small molecules and drug inducers targeting iron metabolism: such as Erastin, Temozolomide (TMZ), and small molecules like MMRi62. (2) Small molecules and drug inducers targeting lipid metabolism: such as the anti-cancer drug sorafenib, and inhibitors of cardiolipin oxidation like XJB-5–131 and JP4–039. (3) Small molecules and drug inducers targeting the GSH/GPX4 axis: such as RSL3 and RSL5, ML162, DPI7, and DPI10. (4) Small molecules and drug inducers targeting the FSP1/CoQ-related pathway: NDP4928, FIN56, etc. (5)Small molecules and drug inducers targeting other pathways include brequinar, dexamethasone, etc. Inhibitors primarily include (1) Small molecule inhibitors that lower iron levels, such as ciclopirox olamine (CPX), deferiprone (DFP), and deferasirox (DFX). (2) Small molecule inhibitors used to reduce lipid peroxidation include Ferrostatin-1 (Fer-1), α-Tocopherol (Vitamin E), SRS15–72B, SRS15–72A, SRS16–80, and SRS16–86, among others. (3) Small molecule inhibitors affecting the GSH/GPX4 axis: such as β-mercaptoethanol (β-ME), 2-amino-5-chloro-N, 3-dimethylbenzamide (CDDO), a triterpenoid compound, etc. Next, let’s summarize a few representative drugs from the list.

### 3.1 Ferroptosis inducers

Erastin, first reported in 2003 (102), was the earliest discovered ferroptosis inducer. It was later confirmed that Erastin binds to and blocks the transport of cysteine by the cystine/glutamate antiporter, leading to intracellular depletion of GSH and triggering iron-dependent cell death. Affinity purification and mass spectrometry analysis have shown that Erastin interacts with voltage-dependent anion channel 2 (VDAC2) (103), reducing the permeability of VDAC2 to the reduced form of nicotinamide adenine dinucleotide (NADH), altering its ion selectivity, and disrupting mitochondrial respiratory chain oxidative phosphorylation (104).

### 3.2 Ras selective lethal compound 3

Ras-selective lethal small molecule 3 (RSL3) was first reported in 2008 (24), and it wasn’t until 2014 that Yang et al. discovered that GPX4 is the target protein of RSL3, revealing this key pathway molecule (105). RSL3 directly inhibits GPX4, leading to an imbalance in the intracellular redox system and triggering ferroptosis. Alongside RSL3, RSL5 was discovered to induce iron-dependent cell death by targeting VDAC (24).

### 3.3 The inhibitor of ferroptosis inhibitor 1

Bersuker et al. discovered that Apoptosis-Inducing Factor Mitochondria-Associated 2(AIFM2) can reduce cellular sensitivity to iron-dependent cell death by decreasing Coenzyme Q10 (CoQ10) level, thereby exhibiting an anti-ferroptotic effect independent of GPX4 (106). It was consequently renamed Ferroptosis Suppressor Protein 1 (FSP1). Meanwhile, Doll et al. found that the inhibitor of FSP1, iFSP1, can induce selective ferroptosis in GPX4 knockout cells overexpressing FSP1. As a novel inducer of ferroptosis, the mechanisms of iFSP1 are worthy of further exploration (74).

### 3.4 Ferroptosis inhibitors 1(Fer-1)

Fer-1 is a ferroptosis inhibitor obtained through high-throughput screening. It captures lipid peroxides through its lipophilic properties, downregulating prostaglandin-endoperoxide synthase 2 (PTGS2), upregulating GPX4, and nuclear factor erythroid 2-like 2 (NFE2L2) (107). Linkermann et al (98) discovered a third-generation Fer compound, SRS-16–86, which has superior plasma stability and stronger inhibition of ferroptosis compared to Fer-1.

### 2.5 Liproxstatin-1(Lip-1)

Lip-1 is a specific inhibitor of LPO, and its mechanism of action in clearing LPO is similar to that of Fer-1. Lip-1 easily stays within the lipid bilayer, and the free radicals formed after clearing LPO can be reduced by other antioxidants in the body through targeted contact. Research indicates that the aromatic amine structure is essential for Liproxstatin class compounds to reduce peroxides (108).

### 2.6 Others

Other ferroptosis inhibitors include iron chelators like deferoxamine (DFO), ciclopirox olamine (CPX), and antioxidants like vitamin E. They primarily function by reducing iron levels and inhibiting oxidative stress to prevent cell death (109–111).

## 4 Ferroptosis and kidney disease

**Figure 6:**
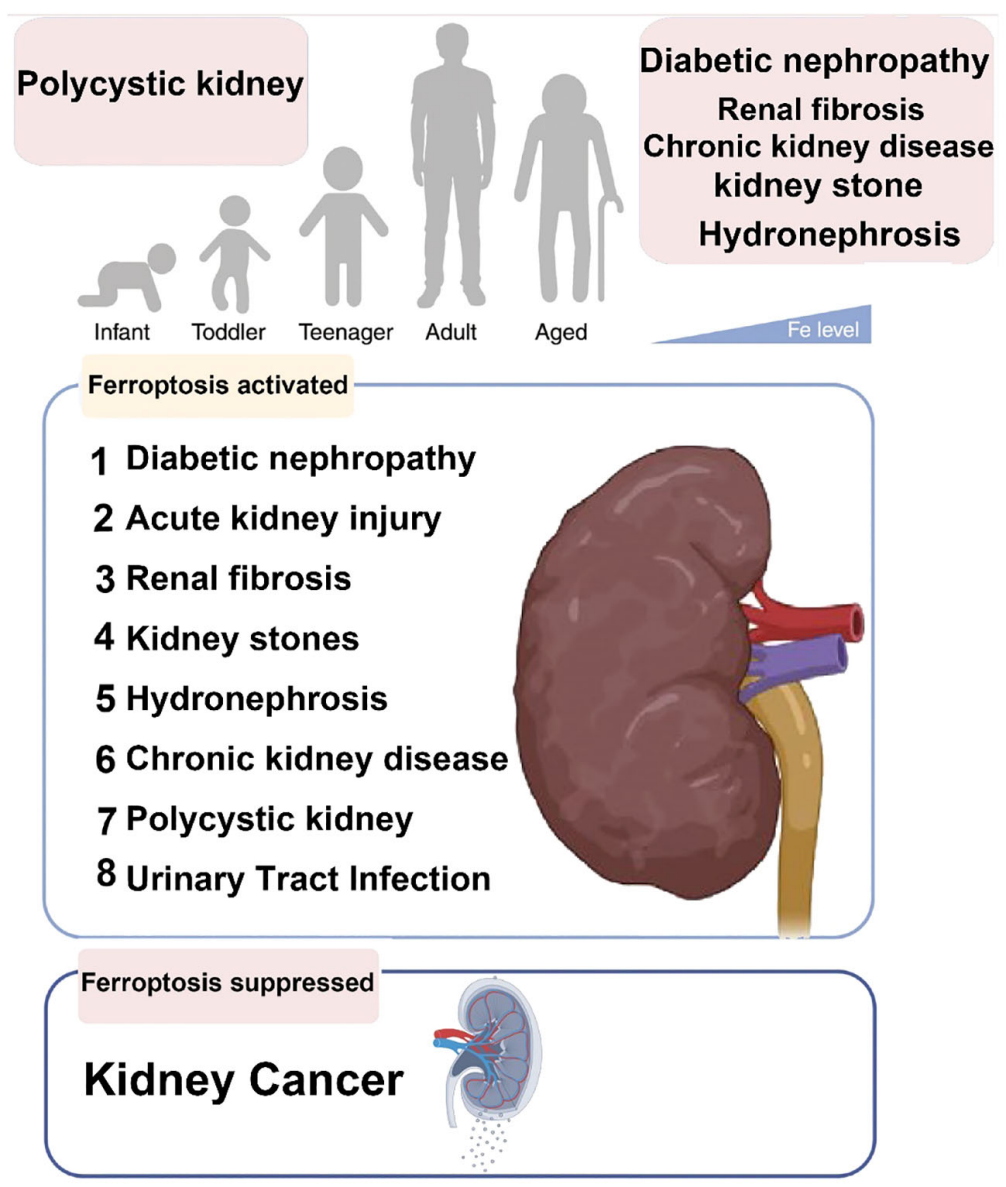
Ferroptosis-related diseases that can present throughout the human lifespan.

Ferroptosis-related diseases that can present throughout the human lifespan are present in **Figure 6**.

### 4.1 Ferroptosis and DN

The pathogenesis of DN involves multiple factors, including dysregulation of glucose and lipid metabolism, oxidative stress, accumulation of advanced glycation end-products (AGEs), inflammation, activation of the innate immune system, genetic susceptibility, activation of the renin-angiotensin system, and miRNAs (112–114).

Recently, researchers have identified hub genes (FPR3, C3AR1, CD14, ITGB2, RAC2, and ITGAM) associated with ferroptosis in diabetic kidney disease through gene differential expression analysis in patients. Non-coding genes (hsa-miR-572, hsa-miR-29a-3p, hsa-miR-29b-3p, hsa-miR-208a-3p, hsa-miR-153–3p, and hsa-miR-29c-3p) and transcription factors (HIF1α, KLF4, KLF5, RUNX1, SP1, VDR, and WT1) that interact with these hub genes may also be relevant to diabetic kidney disease (115). This further deepens our understanding of the molecular mechanisms involved in the pathogenesis of diabetic kidney disease. In patients with type 2 diabetes kidney disease, ferritin levels are elevated, along with significantly increased ROS and MDA levels. The expression of ferroptosis-related proteins ACSL4, PTGS2, and NOX1 is elevated, while GPX4 levels are reduced. In a diabetic kidney disease model, it was observed that the renal tissue shows a significant increase in iron ion content, elevated levels of MDA and 4-hydroxynonenal (4-HNE), decreased GSH, markedly decreased FTH1 expression, significantly increased TFR-1 expression. Transmission electron microscopy revealed mitochondrial membrane rupture, fragmentation, and mitochondrial cristae fragmentation and disappearance. Treatment with Ferrostatin-1 improved the iron overload, accumulation of lipid peroxidation, and antioxidant levels associated with ferroptosis. Additionally, there was a significant decrease in urinary protein, urinary creatinine, and urinary protein/creatinine ratio. This leads to significant reductions in urinary protein, urinary creatinine, and urinary protein/creatinine ratio, along with improvements in glomerular changes, tubular epithelial degeneration with loss of brush border, tubular luminal dilation, folding and fracturing of the tubular basement membrane, renal fibrosis area, and collagen content associated with diabetic kidney disease (116, 117). In high glucose-induced models of renal tubular epithelial cells, mesangial cells, and podocytes, ferroptosis phenomena were also observed. Furthermore, in tubular cells stimulated with TGF-β1, a decrease in GSH concentration and enhanced lipid peroxidation, which are characteristic changes of ferroptosis, were observed.

Ferrostatin-1 was able to alleviate TGF-β1-induced ferroptosis in these cells (117). The above data indicate that ferroptosis contributes to the exacerbation of diabetic kidney injury and kidney fibrosis. Inhibiting ferroptosis may help improve the structure and function of the kidney. Ferroptosis plays a significant role in the advancement of diabetic kidney disease.

#### 4.1.1 The involvement of ferroptosis in the mechanism of DN

Research suggests that ferroptosis leads to the development of DN by inducing injury to renal tubules (117–120), glomeruli (121–126), and kidney fibrosis (127, 128). Tubular injury is a key factor in the development of DN, as high glucose levels trigger renal tubular cell iron overload, reduced antioxidant capacity, excessive ROS production, and lipid peroxidation (129). Animal studies have shown that in the kidneys of mice induced with DN models by streptozotocin and diabetic (db/db) mice, there is an increase in iron content, particularly in the renal tubules. The inhibitor of acyl-CoA synthetase long-chain family 4 (ACSL4), rosiglitazone, improves renal function in DN model mice, reducing lipid peroxidation products and iron content. These effects are related to the alleviation of ferroptosis (118). Mesangial cells in the glomerulus are a special type of smooth muscle cell located between the capillary loops of the glomerular capillaries. Their injury is a fundamental pathological change in DN (123).

Recent studies have indicated that ferroptosis is involved in renal fibrosis in DN (117, 118). The latter represents the ultimate pathological change in DN (130). In patients with DN, serum ferritin, and lactate dehydrogenase release both increase (124); In kidney biopsy specimens, the expression of xCT and GPX4 mRNA decreases (119). A low iron diet or iron chelators can delay the progression of DN in rats (131). Recent studies have suggested that inhibiting ferroptosis may be a novel approach to exploring the progression and treatment of DN (117–119, 124, 131). In conclusion, ferroptosis is involved in the pathogenesis of DN, and targeting the inhibition of ferroptosis holds promise as a new pathway for treating DN.

#### 4.1.2 Targeting the inhibition of ferroptosis for the treatment of DN

Exploration has been conducted on inhibiting ferroptosis as a therapy for DN. Current research indicates that certain active ingredients from natural plant ingredients can target the inhibit ferroptosis, thereby attenuating diabetes-induced tubular and glomerular damage as well as renal fibrosis, ultimately serving as a potential treatment for DN (132–134). Studies suggest that certain drugs or bioactive substances can mediate the occurrence and development of DN by modulating ferroptosis (135). Nobiletin (136) upregulates NRF2, enhancing the antioxidant stress capacity in the kidneys of diabetic mice. Increased expression of FTH-1 and downregulation of TFR-1 help ameliorate iron deposition in the kidneys of diabetic mice. Moreover, pretreatment with nobiletin can reverse mitochondrial morphological changes induced by diabetic ferroptosis, delaying the progression of DN. The ACSL4 inhibitor rosiglitazone (Rosi) can also alleviate diabetic kidney damage by inhibiting ferroptosis (135). The role of certain traditional Chinese herbs in regulating ferroptosis is also noteworthy. Gimatinib inhibits podocyte ferroptosis by modulating the mmu_circRNA_0000309/miR-188–3p/GPX4 signaling axis, thus improving diabetic kidney damage (137). Berberine can significantly improve the levels of ROS and GSH in podocytes induced by high glucose, upregulate NRF2 expression, and thereby alleviate podocyte ferroptosis (138). Furthermore, Aloe-emodin can significantly improve oxidative stress responses, downregulate the expression of HMOX-1 and NRF2, and inhibit ferroptosis levels (139). HMGB1 regulates mesangial cell ferroptosis induced by high glucose through the NRF2 pathway (124). Upregulation of Prdx6 expression mediated by Sp1 can alleviate oxidative stress and ferroptosis, preventing podocyte damage in DN (126). Furthermore, certain endogenous active peptides like salicin-β can promote high glucose-induced ferroptosis in HK-2 cells by regulating gene expression of antioxidant systems (GPX4 and SLC7A1) and iron metabolism regulatory systems (FTH-1 and TFR-1) (129). These studies suggest that in the future, regulating DN from the perspective of iron cell death may provide new insights for the treatment of diabetic kidney disease.

#### 4.1.3 Targeting ferroptosis to improve tubular damage in DN

Ferroptosis mediates tubular injury in DN, suggesting that inhibiting tubular injury ferroptosis may provide a therapeutic approach for DN. Licorice may exert therapeutic effects on diabetes and its complications through anti-inflammatory or antioxidant mechanisms (140). Glabridin, a flavonoid extracted from the natural plant component licorice, can promote tubular epithelial cell survival by increasing the activity of superoxide dismutase (SOD) and GSH in NRK-52E cells, upregulating the expression of GPX4, SLC7A11, and SLC3A2, reducing malondialdehyde and iron concentrations, lowering TfR1 expression to inhibit ferroptosis in DN. Similarly, the flavonoid calycosin, which also possesses antioxidant and anti-inflammatory properties, prevents high glucose-induced cell ferroptotic damage by upregulating the GSH/GPX4 pathway in human renal proximal tubular epithelial cells (HK-2 cells), reducing LPO, and inhibiting the expression of nuclear receptor coactivator 4 (NCOA4) (141). Umbelliferone (142) and platycodin D (143) protect renal tubules by inhibiting iron cell ferroptosis in HK-2 cells and blocking cell damage induced by high glucose. Therefore, the above-mentioned compounds may exert therapeutic effects on DN by inhibiting ferroptosis in renal tubular cells.

#### 4.1.4 Targeting ferroptosis to improve glomerular injury in DN

Mesangial cells are a special type of smooth muscle cell distributed between the capillary loops of glomerular capillaries. Their injury is a basic pathological change in DN renal damage (123). *In vitro*, experimental results demonstrate that the ferroptosis inducers erastin and high glucose both induce ferroptosis in the mesangial cells of the glomeruli. High glucose and erastin significantly induce LDH release, promote the expression of ACSL4, cyclooxygenase 2, and NADPH oxidase 1, and decrease GPX4 levels. Conversely, iron chelators reverse the glucose-induced LDH release and alterations in ferroptosis-related genes in mouse mesangial cells, indicating that high glucose can induce ferroptosis in mesangial cells (124). High glucose can induce ferroptosis in podocytes, leading to podocyte injury (126). Berberine in high glucose-induced podocytes inhibits ROS production, promotes GSH generation, upregulates the expression of nuclear factor-erythroid 2-related factor 2 (Nrf2), heme oxygenase-1 (HO-1), GPX4, and podocin, and decreases the levels of cyclooxygenase 2 and ACSL4. This alleviates podocyte cytoplasmic membrane foaming and mitochondrial shrinkage under high glucose conditions. By activating the Nrf2/HO-1/GPX4 pathway, it inhibits ferroptosis in podocytes, thereby exerting its renal protective effect (144). Glycyrrhizic acid primarily exerts renal protective effects by upregulating GPX4 and inhibiting ROS production to inhibit high glucose-induced iron cell death in podocytes (145). In conclusion, the active ingredients of the aforementioned natural plant ingredients may improve glomerular damage caused by DN by inhibiting ferroptosis.

#### 4.1.5 Targeting ferroptosis to improve renal fibrosis in DN

Studies indicate that ferroptosis is involved in renal fibrosis in DN, which is the final pathological change in DN (130). In a diabetic rat model induced by a high-sugar diet and streptozotocin, Sirius Red staining revealed that liquiritigenin improved kidney function, and inhibited renal interstitial fibrosis. This is related to its promotion of SOD and GSH activity, upregulation of GPX4, SLC7A11, and SLC3A2 expression, reduction of malondialdehyde content and iron concentration, as well as downregulation of TFR1 expression. These findings indicate that liquiritigenin improves renal fibrosis in DN by inhibiting ferroptosis (131). In db/db diabetic mouse models, hesperetin can upregulate GPX4 expression, inhibit LPO and NCOA4 expression, and suppress collagen deposition in renal tissue. This indicates that hesperetin may reduce collagen deposition and renal fibrosis by inhibiting ferroptosis (140). The above-mentioned compounds may improve renal fibrosis in DN by inhibiting ferroptosis.

Since the involvement of ferroptosis in the pathogenesis of DN was identified in 2020, scholars have started to explore drugs targeting the inhibition of ferroptosis for the treatment of DN. Some active ingredients from natural plant components can alleviate tubular and glomerular injury as well as renal fibrosis induced by high glucose and diabetes by selectively inhibiting ferroptosis, thereby serving as a treatment for DN. Although exploratory experimental studies currently indicate that ferroptosis is involved in the pathology of DN and can be targeted and intervened pharmacologically, the detailed mechanism still needs to be elucidated. The broad application prospects also require further elucidation. In conclusion, ferroptosis plays a significant role in the progression of DN, and targeting ferroptosis is a promising therapeutic approach for treating DN. It is a potential treatment approach with hopeful prospects.

### 4.2 Ferroptosis and AKI

AKI has always been a severely debilitating disease worldwide, and it remains a focus of clinical research (146). In recent years, the incidence and mortality rate of AKI has been on the rise. AKI has been associated with acute changes in kidney function and long-term prognosis, including progression to CKD, cardiovascular disease, persistent function, and even death (147, 148). AKI is caused by a variety of factors. Prerenal AKI refers to AKI caused by inadequate renal perfusion. AKI caused by renal parenchymal injury is named based on the location of the injury (glomerular, tubular, or interstitial) (149). The pathophysiological response to AKI may determine whether kidney function is recovered or progresses to CKD. Regeneration of tubular epithelial cells promotes recovery, whereas interstitial fibrosis and loss of renal capillaries are associated with progression to CKD (150). Experimental models have demonstrated that AKI can lead to chronic damage of renal parenchyma, resulting in CKD, indicating that early intervention may impact long-term outcomes (151). However, the specific mechanisms of AKI occurrence and development of AKI are not yet clear. Currently, there are no effective treatment methods to prevent the occurrence of AKI.

#### 4.2.1 AKI caused by ischemia-reperfusion injury

In ischemia-reperfusion injury-induced AKI, apoptosis has long been considered the primary mechanism of cell death. However, using apoptosis-related inhibitors has not been effective in blocking the occurrence of AKI (152). On the contrary, using ferroptosis inhibitors such as liproxstatin-1 has been shown to alleviate tissue damage caused by ischemia-reperfusion and significantly protect kidney function (153). Pannexin-1 is a member of the ATP-release pathway protein family. Research (154) has shown that silencing pannexin-1 can promote the expression of the intracellular antioxidant enzyme HO-1 and inhibit ferroptosis through the mitogen-activated protein kinase/extracellular signal-regulated kinase pathway, thereby reducing ischemia-reperfusion injury in the kidneys. Irisin is an exercise-induced hormone that can improve mitochondrial function and reduce the production of ROS. Research (155) has found that treatment with irisin can significantly alleviate the inflammatory response, endoplasmic reticulum stress, and oxidative stress in mice with renal ischemia-reperfusion injury. Its mechanism of action may be related to the upregulation of GPX4 expression. Quercetin is a natural flavonoid compound known for its pharmacological properties such as antioxidant, anti-inflammatory, and anti-aging effects. Research (156) has shown that quercetin can inhibit ferroptosis in renal tubular epithelial cells by downregulating the expression of activating transcription factor 3 gene, leading to a significant increase in the expression of SLC7A11 and GPX4. This conclusion has been validated by studies on the regulatory role of microRNAs (miRNAs, miR) on ischemia-reperfusion-induced renal injury in rats (157). Ischemia-reperfusion induced upregulation of miR-182–5p and miR-378a-3p, leading to activation of ferroptosis in kidney injury through downregulation of GPX4 and SLC7A11. Therefore, ferroptosis may be the primary pathway through which ischemia-reperfusion injury triggers AKI.

#### 4.2.2 AKI caused by cisplatin

Cisplatin is a widely used anti-tumor drug, and its main adverse effect is severe nephrotoxicity (158). Researchers have long been striving to elucidate the mechanisms underlying cisplatin-induced nephrotoxicity to better utilize this therapeutic drug. GPX4 is significantly downregulated in cisplatin-induced AKI, while ferroptosis biomarkers 4-hydroxynonenal and malondialdehyde are upregulated. This indicates that ferroptosis plays an important role in cisplatin-induced AKI (159). The Vitamin D receptor agonist paricalcitol can prevent cisplatin-induced AKI by reducing lipid peroxidation and reversing GPX4 downregulation (160). This is similar to the mechanism of mangiferin in treating cisplatin-induced AKI (159). Conversely, overexpression of myo-inositol oxygenase in proximal renal tubules can exacerbate ferroptotic damage in the kidneys of mice treated with cisplatin (161). Ras homolog enriched in brain 1 (Rheb1), a GTPase, plays a crucial role in regulating cell growth, differentiation, and survival in the brain. Research (162) found that Rheb1 can prevent cisplatin-induced ferroptosis in renal tubular cells by maintaining mitochondrial homeostasis. Fumarate esters, an oral small molecule drug, have been found in a study (163) to prevent ferroptosis through its antioxidant action via Nrf2 and improve AKI. Furthermore, research (164) has demonstrated that mice with knockout of the ferritin heavy chain gene exhibit more severe kidney injury after cisplatin injection compared to control mice. Indicating the crucial protective role of the ferritin heavy chain as a significant iron metabolism-related protein in renal tubular damage. The above research results also indicate that the use of iron chelators such as deferoxamine or iron suppressors like Ferristatin-1 can significantly alleviate cisplatin-induced acute AKI (161, 164).

#### 4.2.3 Folic acid-induced AKI

The folic acid-induced acute kidney injury (AKI) model is commonly regarded as an excellent model for replicating human AKI. Studies have shown that in mice pre-treated with the ferroptosis inhibitor Ferrostatin-1, folic acid-induced intracellular lipid peroxidation and tissue damage were markedly reduced, leading to improved kidney function (165). FG-4592, a hypoxia-inducible factor-prolyl hydroxylase inhibitor, has been found to elevate intracellular GSH levels and decrease iron accumulation when administered as a pre-treatment (166). Its protective mechanism primarily involves the activation of the intracellular antioxidant enzyme Nrf2, thereby inhibiting folic acid-induced renal cell ferroptosis and slowing down fibrosis progression. Studies have also indicated that nuclear receptor subfamily 1 group D member 1 (NR1D1) can stimulate ferroptosis by directly binding to ROR response elements and repressing the transcription of SLC7A11 and HO-1. Consequently, targeting and inhibiting NR1D1 may restrain ferroptosis, thereby ameliorating folic acid-induced AKI in mice (167). Notably, nuciferine, the primary bioactive compound isolated from lotus leaf, can prevent iron accumulation and lipid peroxidation in folic acid-induced AKI by enhancing intracellular GSH and GPX4 levels, ultimately inhibiting ferroptosis (166). These research findings collectively underscore the significant role of ferroptosis in folic acid-induced AKI.

#### 4.2.4 AKI caused by rhabdomyolysis

The causes of rhabdomyolysis include factors such as trauma, drugs, toxins, and infections, with AKI being a serious complication of rhabdomyolysis. Research (168) indicates that the Fe^2+^ directly induced by myoglobin metabolism may lead to lipid peroxidation in proximal tubule epithelial cells, which could be an important pathogenic mechanism of rhabdomyolysis-induced acute AKI. Guerrero-Hue et al (169) discovered that curcumin, as a potent antioxidant, can inhibit ferroptosis in kidney cells. The mechanism may involve the inhibition of the Toll-like receptor 4/NF-κB signaling pathway and the activation of intracellular HO-1, which reduces the myoglobin-mediated inflammation and oxidative stress response. The study also found that the use of iron chelator-1 significantly improved renal function in glycerol-injected mice.

#### 4.2.5 Other models of AKI

Aristolochic Acid I (AAI) is an important metabolite of aristolochic acid, which has been found to have significant nephrotoxicity. Research has shown that it can significantly decrease the levels of intracellular GSH while simultaneously upregulating the expression of 4-hydroxynonenal and Fe^2+^. Iron chelators such as deferoxamine mesylate and ferroptosis inhibitor ferrostatin-1 have been found to significantly alleviate the cell toxicity induced by aristolochic acid I. The Nrf2/HO-1/GPX4 antioxidant signaling pathway may be an important intervention target for preventing drug-induced AKI containing AAI. AKI (170). Alpha-lipoic acid is a natural antioxidant with the ability to scavenge free radicals and chelate toxic metals. Research (171) has found that it can effectively mitigate cobalt-induced ferroptosis in the kidneys due to metal implants in the human body. The above research results indicate that ferroptosis is widely involved in the occurrence and development mechanisms of various types of AKI.

#### 4.2.6 Targeting ferroptosis for the treatment of AKI

Given that ferroptosis is extensively involved in the occurrence and development mechanisms of various types of AKI, targeting the ferroptosis pathway may be a novel strategy for preventing and treating AKI. This strategy mainly includes iron chelation therapy, targeting iron metabolism-related proteins, lipophilic antioxidants, and direct inhibitors of ferroptosis (172). The overall strategy of iron chelation therapy is to reduce the unstable iron pool, minimize the production of ROS, and thus prevent lipid peroxidation caused by excessive iron overload. In addition to iron chelators, cellular iron depletion can also be achieved by targeting iron metabolism-related proteins. Research has found that increasing the iron regulator hepcidin level in the bloodstream can induce the degradation of ferroportin 1 and promote ferritin expression, effectively restoring iron homeostasis and reducing the generation of ROS (148). Overactivation of intracellular Nrf2 can not only promote the production of a series of downstream antioxidant enzymes but also increase the level of GSH, effectively inhibiting the progression of renal ischemia-reperfusion injury in the early stages (173). HO-1, as an intracellular antioxidant protective enzyme, plays a good role in preventing AKI in various animal injury models induced by ischemia-reperfusion, cisplatin, and lipopolysaccharide (174). Iron-regulatory proteins such as ferritin-1, as representatives of ferroptosis inhibitors, primarily inhibit iron-dependent cell death by interfering with lipid peroxidation (174). However, the *in vivo* use of ferritin-1 is limited by its stability and lower effectiveness. Therefore, there is an urgent need to develop a safer, more stable ferroptosis inhibitor that can be used for treating human diseases in clinical settings.

#### 4.2.7 Natural plant compounds regulate ferroptosis to intervene in kidney injury

Natural plant components offer unique advantages in the prevention and treatment of kidney injury. Compounds such as glycyrrhizic acid, astragaloside IV, ginsenoside Rg1, and dioscin, derived from licorice, astragalus, ginseng, and yam, respectively, possess natural properties that nourish qi, nourish yin, invigorate the spleen, and benefit the lungs. Similarly, compounds like paeoniflorin and curcumin, sourced from peony and turmeric, are known for their natural properties that promote blood circulation and remove blood stasis. Furthermore, compounds such as acteoside and emodin, derived from natural plant sources, are recognized for their heat-clearing and detoxifying effects. These natural components align with the pathogenesis and treatment principles of acute kidney injury (AKI). In recent years, there have been numerous reports on the use of natural plant compounds to regulate ferroptosis and alleviate kidney injury, showcasing targeted therapy and significant efficacy (**Tables 2**, **3**).

**Table 2:** Reagents that modulate ferroptosis (Ferroptosis inhibitors).

Reagents	Targets	Impact on ferroptosis
Ferrostatin-1	Lipid peroxidation	Inhibition of lipid peroxidation
Liproxstatin-1	Lipid peroxidation	Inhibition of lipid peroxidation
Vitamin E	Lipid peroxidation	Inhibition of lipid peroxidation
SRS 16–86、SRS 11–92	Lipid peroxidation	Inhibition of lipid peroxidation
Troglitazone, Pioglitazone, Rosiglitazone	ACSL4	Inactivation of ACSL4
Deuterated polyunsaturated fatty acids (D-PUFAs)	Lipid peroxidation	Inhibition of lipid peroxidation
XJB-5–131	Lipid peroxidation	Inhibition of lipid peroxidation
Butylated hydroxytoluene, butylated	Lipid peroxidation	Inhibition of lipid peroxidation
hydroxyanisole	Lipid peroxidation	Inhibition of lipid peroxidation
Ferrostatins, liproxstatins	Lipid peroxidation	Inhibition of lipid peroxidation
CDC, PD-146176, AA-861, zileuton	Lipoxygenase	Inhibition of lipid peroxidation
Selenium	Selenoproteins	Inhibition of lipid peroxidation
Deferoxamine, cyclipirox, deferiprone	Intracellular iron	Decreases cellular iron
Vildagliptin, alogliptin, and Linagliptin	DPP4	Blocks DPP4-mediated lipid peroxidation
Irisin	GPX4	Upregulates GPX4
Melatonin	System Xc and GPX4-	Upregulates system Xc and GPX4-
Vitexin	System Xc and GPX4-	Upregulates system Xc and GPX4-
Isoliquiritigenin	System Xc and GPX4-	Upregulates system Xc and GPX4-
Vitamin A	Lipid peroxidation	Blocks lipid peroxidation
Paricalcitol	GPX4	Upregulates GPX4
Pachymic acid	Nrf2,GPX4, SCL7A11, and HO-	Upregulates of Nrf2, GPX4, SCL7A11 and HO-1
Rheb1	Maintains mitochondrial homeostasis	Maintains mitochondrial homeostasis
Quercetin	System Xc and GPX4-	Upregulates system Xc and GPX4-
Artesunate	Lipoxygenases	Blocks lipid peroxidation

**Table 3:** Reagents that modulate ferroptosis (Ferroptosis inducers).

Reagents	Targets	Impact on ferroptosis
Sulphasalazine	System X_c_^-^	Prevents cystine import, causes GSH depletion
Sorafenib	System X_c_^-^	Prevents cystine import, causes GSH depletion
Glutamate	System X_c_^-^	Prevents cystine import, causes GSH depletion
Erastin and its analogs	System X_c_^-^ VDAC2/3-	Prevents cystine import, causes GSH depletion
RSL3	GPX4	Covalent inhibitor of GPX4 that causes accumulation of lipid hydroperoxides
ML162	GPX4	Covalent inhibitor of GPX4 that causes accumulation of lipid hydroperoxides
FINO_2_	GPX4	Covalent inhibitor of GPX4 that causes accumulation of lipid hydroperoxides
FIN56	CoQ10 and GPX4	Depletes CoQ10 via SQS-mevalonate pathway and causes a decrease in GPX4 protein abundance
BSO, DPI2, cisplatin	GHS	GHS deletion
Statins (e.g., cerivastatin, simvastatin)	HMGCR	Blocks CoQ10 biosynthesis
Trigonelline, brusatol	Nrf2	Nrf2 inhibition
Siramesine, lapatinib	Ferroportin, Transferrin	Upregulates cellular iron
Brequinar	DHODH	Inhibits DHODH
iFSP	FSP1	Inhibits FSP1
BAY 87‐2243	Mitochondrial respiratory chain	Inhibits mitochondrial respiratory chain
Andrographolide	Lipid peroxidation	Promotes lipid peroxidation
Toosendanin	Lipid peroxidation	Promotes lipid peroxidation
Gliotoxin	ROS, lipid peroxidation	Promotes lipid peroxidation
Arsenic trioxide	ACSL4	ACSL4 activation
Manganese	ROS, lipid peroxidation	Promotes lipid peroxidation
Fatostatin	GPX4	Inhibits GPX4 expression
Legumain	GPX4	Facilitates chaperone-mediated autophagy of GPX4
Dihydroartemisinin (DHA)	Ferritin, iron overload	Increases cellular iron
Artemisinins	Iron overload	Increases cellular iron

(1) Flavonoids: Baicalein mainly exists in the roots of Scutellaria baicalensis and Scutellaria lateriflora. being one of the flavonoids with the highest content in Scutellaria baicalensis. It has effects such as reducing cerebral vascular resistance, anti-inflammatory, and antibacterial properties (175). Research has found that baicalein has significant anti-ferroptosis activity. It markedly inhibits GPX4 degradation, and lipid peroxidation, and enhances cellular resistance to ferroptosis. In the AKI model induced by polymyxin B (PMB), baicalein reduces P53 acetylation levels, inhibits ferroptosis, and ultimately alleviates AKI (175). Isoliquiritigenin is an isoflavone compound found in licorice, with various pharmacological effects such as anti-tumor, antioxidant, and anti-inflammatory properties. It can inhibit the expression of HMGB1 and NCOA4 induced by lipopolysaccharides (LPS), suppress the accumulation of free iron in renal tubular epithelial cells, alleviate mitochondrial damage in renal tubules, enhance the expression levels of GPX4, and provide certain protective effects on kidney function (176). Chrysanthemin-3-glucose has antioxidant and anti-tumor effects. It significantly reduces levels of Fe^2+^, ROS, MDA, and ACSL4 in AKI mice and damaged renal tubular epithelial cells By activating the AMPK pathway. It also increases GPX4 and GSH levels, effectively inhibiting ferroptosis and alleviating kidney damage (177).
(2) Saponins: Astragaloside IV, an active component of Astragalus, activates the PI3K/AKT and Nrf2 signaling pathways, reduces oxidative stress, enhances GPX4 and Nrf2 expression, reduces iron accumulation, inhibits ferroptosis induced by Aflatoxin, significantly improves kidney damage, and protects kidney cells (178). Ginsenoside Rg1, a compound found in Panax ginseng, exhibits a positive therapeutic effect on kidney diseases. It promotes the expression of FSP1, reduces cellular level of Fe^2+^, ferritin heavy chain, and MDA, increases GPX4, and GSH, inhibits lipid peroxidation and cell ferroptosis, enhances the vitality of renal tubular cells, and alleviates AKI in septic rats (179). Diosgenin saponin, as the basic raw material for synthesizing various steroidal hormones, is present in natural plants such as Dioscoreaceae and Fabaceae. It possesses pharmacological properties such as anti-inflammatory, anti-tumor, and antioxidant effects. Research indicates that it can alleviate kidney tissue lesions and mitochondrial damage in mice, reducing inflammatory responses (180); Furthermore, by promoting Nrf2 expression and activating HO-1, it significantly reduces ROS levels and MDA content in the kidneys of AKI rats, increases levels of GSH, GPX4, and other anti-ferroptosis proteins, markedly reduces the number of apoptotic cells in the kidneys and renal tubular epithelial cells of model rats, and promote the expression levels of pro-apoptotic proteins, thereby protecting the kidneys (181). Paeoniflorin possesses pharmacological properties such as analgesic, sedative, vasodilatory, antipyretic, and anti-inflammatory effects. It plays a beneficial role in protecting kidney diseases such as AKI and DN. *In vitro* experiments have shown that paeoniflorin can upregulate the expression levels of SLC7A11, thereby inhibiting ferroptosis in renal tubular epithelial cells, providing a potential therapeutic strategy for protecting kidney tissues (182).
(3) Alkaloids: Nelumbine, a bioactive alkaloid isolated from lotus leaf, exhibits strong antioxidant properties and targets the suppression of folate-induced ferroptosis-related renal pathological changes by regulating the levels of GPX4, SLC7A11, and FSP1. It also improves inflammation, cell infiltration, and kidney function impairment in renal tubular epithelial cells (166). Protopine is a substance extracted from the fresh or dried aerial parts of motherwort. By activating the Nrf2 pathway, it disrupts iron accumulation, lipid peroxidation, and ferroptosis, preventing the downregulation of GSH and GPX4 levels, thus ameliorating cisplatin-induced AKI (183).
(4) Polyphenols: Rhein, as one of the main components of rhubarb, has been proven to possess antibacterial and antioxidant effects. Recent research has shown that rhein can alleviate changes in the renal microstructure of model rats, and decrease the expression of apoptosis-related proteins, thereby reducing cell apoptosis, and counteracting the damage caused by oxidative stress to the kidneys (184). Further research reveals that rhein can alleviate endoplasmic reticulum stress induced by H/R, upregulate GPX4 and SLC7A11 to mitigate lipid peroxidation, reduce renal ferroptosis, and protect against AKI (185). Curcumin is a lipophilic polyphenol extracted from the rhizomes of ginger and turmeric plants. It possesses renal protective properties by inhibiting the TLR4/NF-κB signaling pathway, activating HO-1 to suppress myoglobin-induced inflammation and oxidative stress. This action helps improve lipid peroxidation, decreased antioxidant capacity, and myoglobin-induced ferroptosis in renal tubular cells, thereby alleviating kidney damage (169). Tiger cane glucoside is derived from the dried rhizomes of the Polygonum cuspidatum, a plant in the Polygonaceae family. It possesses various pharmacological activities such as anti-inflammatory and antioxidant effects, and has a protective effect on AKI (186). It significantly reduces the excessive production of free iron, ROS, and MDA induced by cisplatin, alleviates GSH depletion, and distinctly reverses ferroptosis in renal tubular epithelial cells, thereby offering protection to the kidneys (187).
(5) Triterpenoids: Centelloside is a triterpenoid compound isolated from gotu kola, possessing anti-inflammatory and antioxidant properties. Research has shown that centelloside selectively regulates the Nrf2 pathway, increasing the expression of Nrf2, GPX4, and SLC7A11 in a kidney injury model, inhibiting oxidative stress and ferroptosis, alleviating LPS-induced vacuolation and glomerular mesangial expansion in kidney tissues, to reduce AKI (188). Poria acid has anti-renal interstitial fibrosis and can improve renal pathological damage. It activates the Nrf2 signaling pathway, decreases MDA and cyclooxygenase-2 (COX-2) expression, increases GSH, GPX4, and SLC7A11, inhibits blood creatinine and urea nitrogen retention caused by renal ischemia-reperfusion injury, thus ameliorating renal pathology (189). In conclusion, ferroptosis is involved in the pathogenesis of AKI, and natural plant components can alleviate ferroptosis, and reduce AKI progression by targeting the inhibition of GPX4 degradation and lipid peroxidation. However, there is currently no reported evidence on whether traditional Chinese medicines and herbal formulations can also alleviate AKI by inhibiting ferroptosis. With the advancement of science and technology, further exploration of the deep mechanisms of natural plant components in regulating AKI ferroptosis can be carried out through various techniques such as mass spectrometry analysis, metabolomics, and genomics.

In summary, ferroptosis, as a novel form of cell death, is involved in the pathogenesis of various diseases. It plays an indispensable role in the pathogenesis of AKI. Due to the incomplete understanding of the specific mechanisms of ferroptosis in AKI and the high mortality rate associated with AKI, exploring therapeutic targets for AKI from the perspective of ferroptosis may represent a novel direction with great potential for development. In recent years, there has been an increasing amount of research on using natural plant compounds to inhibit ferroptosis and alleviate kidney damage. Natural plant components are complex, and future studies could focus on enhancing the activity of GPX4 and System X_c_^-^, inhibiting lipid peroxidation, and targeted regulation of the Nrf2 signaling pathway to reduce ferroptosis in the kidney. However, most studies have focused on the active ingredients of natural plant compounds, failing to fully harness the complex effects of these compounds. Additionally, the long-term therapeutic efficacy of active ingredients from natural plant compounds has not been fully evaluated yet. This review focuses on intervening in AKI by targeting ferroptosis and summarizes the targets in traditional Chinese medicine for regulating ferroptosis in AKI. However, there is currently limited research on complex formulas, and the mechanisms of intervening in AKI through ferroptosis have not been deeply explored. In the future, natural plant compounds could enhance the therapeutic effects on ferroptosis in AKI by targeting pathways, receptors, inhibitors, activators, and other methods. This could further promote the development of traditional Chinese medicine in the future.

### 4.3 The relationship between ferroptosis and renal fibrosis

Renal fibrosis is the ultimate common process and main pathological manifestation of CKD caused by different etiologies, characterized primarily by the activation of myofibroblasts. The transforming growth factor-β (TGF-β)/Smad signaling pathway is a major pathway in renal fibrosis, and it is closely related to ferroptosis (190). TGF-β is a major pro-fibrotic factor, and various factors can promote its secretion, such as angiotensin II, hypoxia-inducible factor 1, mitogen-activated protein kinase, and high glucose (190). Research indicates that TGF-β1 modulates renal injury by stimulating downstream Smads, where Smad3 is a key mediator that promotes renal fibrosis, Smad2, and Smad7 have renal protective effects, and Smad4 exhibits both promotion of fibrosis and inhibition of inflammation through different mechanisms (191). additionally, Smads also interact with other signaling pathways such as the mitogen-activated protein kinase and nuclear factor κB pathways to positively or negatively regulate renal inflammation and fibrosis (192). TGF-β secreted by tumor vascular endothelial cells can induce differentiation of endothelial cells and adjacent fibroblasts into myofibroblasts through both autocrine and paracrine mechanisms (193). Renal tubular epithelial cells can release exosomes containing TGF-β mRNA to activate fibroblasts, thereby promoting renal fibrosis following AKI (194). Research has confirmed that the pro-fibrotic factor Wnt-1-induced signaling protein 1 (WISP-1) may mediate renal fibrosis by enhancing autophagy mediated by TGF-β1 (195, 196). The physiological functions of iron include participating in the mitochondrial respiratory chain and hemoglobin synthesis. Both iron excess and deficiency are detrimental to metabolic homeostasis. Iron excess can lead to the generation of free radicals, and it may result in organelle stress and disruption of cellular structural integrity. Renal cells are susceptible to the effects of iron overload, and tissue iron deposition can lead to oxidative damage and pathological reactions, including fibrosis and inflammatory reactions (197). Ferroptosis is a non-traditional form of RCD characterized by iron overload and lipid peroxidation. Erastin, Sorafenib, RSL3, and FIN56 are common inducers of ferroptosis, while deferoxamine, Ferrostatin-1 (Fer-1), and Liproxstatin-1 (Lip-1) are common inhibitors of this process. Obstruction of the ureter leads to ferroptosis in renal tubular epithelial cells, which in turn causes the secretion of various fibrogenic factors and affects interstitial fibroblasts in a paracrine manner, promoting their proliferation and differentiation (128). However, the specific mechanism of fibrogenic factor secretion during ferroptosis in renal tubular epithelial cells remains unclear. In TGF-β1-stimulated renal tubular epithelial cell injury, the expression of SLC7A11 and GPx4 is decreased, while Fer-1 can alleviate this change (198).

Inhibition of ferroptosis to alleviate renal fibrosis: Studies indicate that targeting ferroptosis specifically with certain inhibitors can mitigate kidney damage and renal fibrosis: (1) In a 5/6 nephrectomy-induced CKD rat model, deferoxamine alleviates kidney injury and fibrosis by regulating iron metabolism and the TGF-β1/Smad3 pathway (199). (2) Lip-1 significantly attenuates renal collagen protein deposition and expression of fibrotic factors in a unilateral ureteral occlusion (UUO) mouse model, thereby alleviating renal fibrosis. Moreover, it reduces the activation of surrounding fibroblasts by inhibiting the paracrine secretion of fibrosis-promoting fibrotic factors in human renal proximal tubule cells (HK2) (200). (3) Fer-1 and deferiprone can inhibit iron-dependent cell death in renal tubular epithelial cells, thereby reducing kidney injury and fibrosis induced by UUO or ischemia-reperfusion (128). (4) Berberine reduces lipid peroxidation and inhibits ferroptosis by activating adenosine monophosphate-activated protein kinase in a renal ischemia-reperfusion mouse model, thereby alleviating kidney fibrosis (201). (5) Irisin can inhibit ferroptosis induced by Erastin/RSL3 and fibrosis stimulated by TGF-β1 in primary renal tubular epithelial cells. Irisin also significantly alleviates tubular epithelial cell injury and fibrosis by inhibiting Smad3 phosphorylation and suppressing the expression of Nox4 (a downstream regulator of ferroptosis) in the UUO mouse model, thus blocking Smad3-mediated ferroptosis (202). Furthermore, Balzer et al. established an adaptive repair and maladaptation (fibrosis) kidney regeneration model by titrating ischemic injury doses (203). Through detailed biochemical and histological analysis of a maladaptive/pro-fibrotic cluster of proximal tubules, they discovered that cell necroptosis/ferroptosis is a vulnerable pathway in these pro-fibrotic cells. Pharmacological targeting of cell necroptosis/ferroptosis could promote cell adaptive repair and improve fibrosis. The above studies all indicate that targeting ferroptosis for treatment may prevent renal fibrosis in CKD patients, which shows promising potential applications.

#### 4.3.1 Ferroptosis and fibrosis induced by kidney stones

Kidney stones are a common urinary system disorder, with an incidence rate as high as 14.8% and a recurrence rate of up to 50%. 0.8% to 17.5% of kidney stone patients also suffer from CKD. Research has found that genes related to renal interstitial fibrosis are significantly upregulated in patients with kidney stones. Calcium oxalate (CaOx) crystals can adhere to and aggregate on the renal tubular epithelial cells, causing damage to the tubular epithelial cells and endothelial-mesenchymal transition, ultimately leading to renal fibrosis (204, 205). Research has shown that in mice with high oxalate urine and oxalate-stimulated HK-2 cells, there is a significant increase in ROS levels (206), indicating that ferroptosis may be involved in the formation of kidney stones. Furthermore, recent studies have found that CaOx crystals can induce ferroptosis in renal tubular epithelial cells, leading to damage of the tubular epithelial cells and kidney injury. Research has found that exposing renal tubular epithelial cells to various concentrations of CaOx crystal solution results in a significant increase in the expression of ferroptosis-related proteins such as p53, ACSL4, transferrin receptor (TRC), and TF as the concentration of CaOx crystals increases. Conversely, the expression of ferroptosis-inhibiting proteins, solute carrier family 7 member 11 (SLC7A11), and GPX4 decreases relatively. Additionally, due to the stimulation by CaOx crystals, mitochondrial function is impaired and cannot exert antioxidant capabilities, leading to the accumulation of ROS within cells, ultimately inducing ferroptosis. The use of a ferroptosis inhibitor, Ferrostatin-1, can suppress ferroptosis, thereby alleviating damage to renal tubular epithelial cells (207). Furthermore, Song et al (208) found in a cell model of oxalate-induced kidney stones that CaOx crystals can induce ferroptosis through autophagy, thereby exacerbating kidney damage. BECN1 is a molecule that regulates autophagic activity. Experiments have shown that overexpression of BECN1 significantly increases levels of malondialdehyde (MDA), Fe^2+^, and ROS in cells, promoting the occurrence of ferroptosis induced by CaOx crystals. Nuclear receptor coactivator 4 (NCOA4) is a widely expressed intracellular protein that can mediate ferritinophagy to control the release and storage of iron within cells. Studies have shown that knocking out the NCOA4 gene inhibits ferritin degradation and even reverses the effects of BECN1. Other studies have demonstrated that ferroptosis not only participates in cell damage caused by kidney stones but also plays a role in the process of kidney injury repair. Persistent damage to renal tubular epithelial cells can lead to the accumulation of inflammatory proximal tubular cells. These inflammatory proximal tubular cells can significantly downregulate GSH metabolic genes, making cells more prone to ferroptosis, resulting in repair failure after kidney injury, ultimately promoting kidney fibrosis and the progression of AKI to CKD (209). Although ferroptosis is currently believed to be associated with the progression of kidney fibrosis induced by kidney stones, the specific mechanisms remain to be further explored.

#### 4.3.2 Ferroptosis and renal hydronephrosis

Renal hydronephrosis refers to the obstruction of urine drainage from the renal pelvis, leading to an accumulation of urine and increased pressure within the kidney, resulting in the dilation of the renal pelvis and calyces, renal parenchymal atrophy, and a decline in kidney function. Research has found that renal hydronephrosis not only leads to kidney damage but also induces renal fibrosis. Moreover, the severity of renal fibrosis is positively correlated with the severity of renal hydronephrosis (210, 211). At the same time, research indicates that ferroptosis is involved in the process of renal fibrosis caused by renal hydronephrosis. Zhang et al (200) found that in a mouse model of UUO-induced renal hydronephrosis, renal tubular epithelial cells underwent ferroptosis. Additionally, *in vitro* studies suggest that the ferroptosis inhibitor Lip-1 can alleviate renal fibrosis and extracellular matrix deposition. The mechanism may be that Lip-1 inhibits the secretion of pro-fibrotic factors by renal tubular epithelial cells, ultimately suppressing the activation of fibroblasts surrounding the renal tubules. Smad3 is a common signaling protein that can directly promote the expression of pro-fibrotic genes as a transcription factor. Recent studies have revealed that in the mouse model of renal hydronephrosis induced by UUO, the expression of the ferroptosis biomarker GPX4 significantly decreases, accompanied by abnormal activation of Smad3, thereby promoting fibrosis. Additionally, quercetin, as an inhibitor of Smad3 phosphorylation, may inhibit renal interstitial fibrosis in UUO mice by inhibiting Smad3-mediated ferroptosis (202), further confirming the close relationship between ferroptosis and renal fibrosis induced by renal hydronephrosis.

#### 4.3.3 Ferroptosis and CKD

CKD affects 8% to 16% of the global population (212). CKD is defined as a clinical syndrome caused by various factors, characterized by a glomerular filtration rate <60 ml·min-1·(1.73 m2)-1, urine protein >30 mg/d, or the presence of kidney damage markers for more than 3 months. The most common causes are diabetes and hypertension. Zhou et al. (128) found typical ferroptosis characteristics in a CKD mouse model, with decreased expression of GPX4 and increased abundance of 4-HNE. Treatment with Fer-1, which inhibits ferroptosis, reduced kidney damage and fibrosis in the mice. In addition, Jin and Chen (142) found that in diabetic kidney disease, umbelliferone inhibits ferroptosis by activating the nuclear factor E2-related factor 2 (NRF-2)/heme oxygenase-1 (HO-1) pathway, significantly improving kidney damage and reducing ROS generation. In Wang et al.’s study (213) on a rat CKD model, typical ferroptosis characteristics were observed in the CKD group, including increased iron content, oxidative stress, and lipid peroxidation. Further investigation revealed the upregulation of NCOA4 expression and downregulation of FTH1 and FTL expression in residual kidney tissue. Treatment with DFO reversed these phenomena, indicating that ferroptosis in CKD is associated with iron overload caused by ferritin autophagy. Moreover, cellular autophagy is closely related to the progression of CKD to renal fibrosis (214).

CKD is the common outcome of various primary and secondary kidney diseases, characterized by renal interstitial fibrosis and progressive decline in kidney function (215). Studies have shown that the majority of CKD patients experience varying degrees of iron metabolism and lipid metabolism disorders. Disruptions in iron metabolism lead to iron deposition in the kidneys, thereby inducing ferroptosis; disruptions in lipid metabolism result in lipid deposition in the renal parenchyma, promoting lipid peroxidation, further inducing ferroptosis (216). Furthermore, iron-mediated epithelial cell death promotes the secretion of various pro-fibrotic factors such as TGF-β, CTGF, etc., inducing proliferation and differentiation of interstitial fibroblasts, ultimately leading to renal fibrosis. Nait et al. (217) found that the iron chelator deferoxamine can inhibit pathways such as TGF-β1/Smad3, inflammatory response, and oxidative stress, thereby alleviating renal fibrosis in CKD rats. Disruption of iron metabolism and lipid metabolism in CKD can lead to ferroptosis, aggravating renal damage. Targeted therapies focusing on inhibiting ferroptosis may play an important role in protecting renal function in CKD patients.

The above studies indicate that the progression of CKD is closely associated with ferroptosis. By inhibiting ferritinophagy to reduce intracellular free iron levels, thereby suppressing the occurrence of ferroptosis, may emerge as a therapeutic target for CKD.

#### 4.3.4 Targeted inhibition of ferroptosis to alleviate renal fibrosis: key mechanisms

GPX (Glutathione Peroxidase) is a selenoprotein antioxidant enzyme that converts hydrogen peroxide to water (H2O) using oxidized GSH as a substrate. GPX consists of 8 members, among which GPx4 is one of the most important antioxidant enzymes in mammals, regulating the occurrence and progression of ferroptosis (218–220). Renal tubular epithelial cells preferentially oxidize fatty acids as an important energy source (221).

Under normal circumstances, there is a dynamic balance between fatty acid synthesis and oxidation, preventing intracellular lipid accumulation. However, blocking fatty acid oxidation (FAO) in renal tubular epithelial cells can promote intracellular lipid deposition during the fibrotic process. TGF-β can reduce FAO and enhance lipid accumulation associated with renal fibrosis. Research has found that TGF-β1-stimulated renal tubular epithelial cells exhibit increased lipid peroxidation associated with renal failure, a process that can be reversed by GPx4 (153). Leonarduzzi et al. (222) observed that a lack of GPx4 can promote the production of TGF-β1, thereby exacerbating fibrosis, while upregulation of GPx4 can reverse this change. Therefore, the deficiency in GPx4 may be involved in the occurrence and development of renal fibrosis. Conversely, elevation of GPx4 can weaken the activation of the nuclear factor κB pathway, thus alleviating renal fibrosis (223). Interleukin-6 is a pleiotropic cytokine positively correlated with renal fibrosis. Overexpression of GPx4 in fibroblasts can inhibit ultraviolet A radiation-induced release of interleukin-6 (224). In renal biopsy tissue studies of CKD patients and mice models including UUO and renal ischemia-reperfusion injury, downregulation of GPx4 and upregulation of 4-hydroxynonenal were observed. This study demonstrates the potential role and mechanism of ferroptosis in renal tubular epithelial cell death in renal fibrosis. The above conclusions all indicate that GPx4 plays a protective role in the development of renal fibrosis. On the other hand, Lip-1 can inhibit the downregulation of GPX4 expression, and reduce iron deposition and lipid peroxidation, thereby inhibiting ferroptosis. Gong et al (225) found that Erastin promotes the differentiation of fibroblasts into myofibroblasts by increasing lipid peroxidation and inhibiting GPx4 expression, while Fer-1 inhibits ferroptosis and fibrosis by suppressing lipid peroxidation and enhancing GPx4 expression. The above studies all confirm that GPx4 is a key substance for targeted inhibition to alleviate ferroptosis and renal fibrosis. This holds important implications for the specific treatment of renal fibrosis in the future, but further research is still needed to reveal the mechanisms of certain key steps in this process.

In summary, we can observe that in recent years, there has been a considerable amount of evidence supporting the role of ferroptosis in renal fibrosis. This article primarily reviews the main signaling pathways and regulatory factors involved in regulating ferroptosis, the role of ferroptosis in renal fibrosis, and potential therapeutic strategies that interfere with ferroptosis in the treatment of renal fibrosis. While progress has been made, there are still some issues that need to be addressed, including (1) Ferroptosis is not an isolated event and appears to be closely related to other forms of cell death. Besides ferroptosis, necrosis, apoptosis, pyroptosis, autophagy, and other cell death pathways have also been observed in renal fibrosis. Do these cell death mechanisms interact with each other during the fibrosis process? Therefore, studying the potential antagonistic or synergistic effects of ferroptosis in the context of kidney disease is necessary; (2) Currently, research on ferroptosis is based on various disease models, and the impact of ferroptosis on renal fibrosis under physiological conditions is not yet clear; (3) Although regulatory factors of ferroptosis such as ROS, GPx4, GSH, and iron metabolism have been described, they do not constitute suitable sensitive biomarkers for monitoring ferroptosis. Finding simple and reliable biomarkers, especially in the context of kidney disease, remains a challenge; (4) Although the connection between ferroptosis and AKI has been extensively explored, research on the relationship between ferroptosis and renal fibrosis as well as CKD remains limited. Most relevant studies conducted so far have utilized *in vitro* cell culture models or animal models of kidney disease. However, the use of different disease models leads to variations in research outcomes, posing a challenge for the translation of research on the relationship between ferroptosis and renal fibrosis into clinical applications.

### 4.4 Polycystic kidney disease (PKD) and urinary tract infections

PKD is an autosomal dominant genetic kidney disease characterized by the progressive development of cysts in renal epithelial cells, ultimately leading to end-stage renal disease (ESRD). Studies have shown that the mRNA, protein content, and activity levels of antioxidant enzymes such as GPX and superoxide dismutase are downregulated in PKD, resulting in an exacerbation of lipid peroxidation reactions, implicating oxidative stress in the growth of PKD cysts. The Cystic Fibrosis Transmembrane Conductance Regulator (CFTR), a cAMP-activated ATP-gated chloride ion channel expressed in the apical membrane of various epithelial cells, plays a significant role in mediating the efflux of glutathione (GSH) in renal cell lines. In proximal tubule cells, CFTR-mediated reactive oxygen species (ROS)-induced cell death has been observed (226). Simos et al. (227) demonstrated that increased ROS in the cytoplasm activate transmembrane protein 16F (TMEM16F), leading to outward chloride ion flux and disruption of membrane phospholipids. This disruption results in the translocation of phosphatidylserine from the cytoplasm to the extracellular space, potentially leading to sustained lipid peroxidation of the membrane lipids and ultimately cell death, with CFTR playing a synergistic role in this process. Schreiber et al. (228) found that membrane phospholipid peroxidation activates renal TMEM16A, thereby stimulating CFTR. The ferroptosis inhibitor Fer-1 shows promise in preventing the growth of renal cysts, with related clinical trials currently underway. The aforementioned findings suggest that ferroptosis may be one of the pathogenic mechanisms of PKD, and inhibitors of ferroptosis offer a new perspective for developing treatment strategies for PKD (229). Other animal models in chronic kidney disease (CKD) research also provide functional evidence of iron exposure in kidney injury. For instance, in proteinuric CKD rats, restricting iron intake can reduce proteinuria, glomerular iron deposition, and glomerulosclerosis. In the unilateral ureteral obstruction (UUO) animal model, the administration of deferoxamine (DFO) has been shown to alleviate tubulointerstitial fibrosis. Iron chelators can specifically reduce the deposition of iron in the lysosomes of proximal tubules (127). Kidney biopsy specimens from CKD patients reveal the accumulation of iron in renal tubular epithelial cells and infiltrating macrophages. Iron-induced cell death, serving as a trigger, may contribute to the transition from acute kidney injury (AKI) to CKD through sustained oxidative stress and mitochondrial dysfunction. While the kidneys have the function of secreting erythropoietin (EPO), many CKD patients exhibit reduced EPO secretion, leading to a situation where anemia and iron deficiency coexist. Therefore, simply reducing iron exposure is not an effective method for managing CKD, and maintaining iron balance should be approached cautiously.

Urinary tract infection is one of the most common infections in the community and healthcare systems (230), with over 25% of urinary tract infections recurring, leading to the persistence of drug-resistant strains (231). Research indicates that uropathogenic E. coli (UPEC) infecting bladder epithelial cells can activate ferritinophagy when treated with ammonium ferric citrate, leading to the prolonged presence of UPEC in bladder epithelial cells. This increases the risk of recurrent infections and reinfections. In addition to activating ferritinophagy, treatment with ferric ammonium citrate also promotes host bladder epithelial cell death. Knocking out NCOA4 to inhibit ferritinophagy reduces bacterial load and decreases bladder epithelial cell death. Further research reveals that this type of cell death is neither apoptosis nor necrosis but a form of cell death induced by iron overload. However, the authors did not perform specific tests to characterize the phenotype of ferroptosis (232). In conclusion, the persistent presence of UPEC in bladder epithelial cells involves ferritinophagy, which promotes bladder epithelial cell death, exacerbating the severity of urinary tract infections. By inhibiting ferritinophagy and reducing iron content within bladder epithelial cells, it may serve as a potential therapeutic target to control UPEC proliferation. This could provide a theoretical basis for preventing recurrent urinary tract infections and reinfections.

### 4.5 The molecular mechanisms of ferroptosis involvement in renal cell carcinoma

Renal cell carcinoma, abbreviated as RCC, is the most common malignant tumor of the kidney, accounting for 85% to 90% of renal malignant tumors. It can be classified into different subtypes based on pathology, including clear cell carcinoma, chromophobe carcinoma, renal papillary cell carcinoma, medullary carcinoma, and undifferentiated carcinoma (233). According to epidemiological data, the incidence of renal cell carcinoma is second only to bladder tumors and shows an increasing trend year by year (234). The primary treatment methods for renal cell carcinoma include surgical intervention, as well as radiation therapy and chemotherapy. Although there have been some advances in the treatment of renal cell carcinoma in recent years, the prognosis remains suboptimal. Hence, there is an urgent need to explore new targets for its treatment. Numerous studies indicate that inducing ferroptosis in cells may be a novel direction for the treatment of renal cell carcinoma. Yang et al. (235) tested the sensitivity of 117 cancer cell lines to Erastin-induced ferroptosis and found that diffuse large B-cell lymphoma and clear cell renal cell carcinoma (ccRCC) were particularly sensitive to GPX4-regulated ferroptosis. Zou et al. (236) found that GPX4 inhibitors exhibited strong cytotoxicity against ccRCC, with the reduction of GPX4 being a key factor in the occurrence of ferroptosis. Their further investigation revealed that in renal cancer cells, high expression of the hypoxia-inducible factor (HIF) pathway’s hypoxia-inducible factor 2α enhances the sensitivity of ccRCC to ferroptosis by enriching unsaturated fatty acids through hilpda. The von Hippel-Lindau (VHL) gene acts as a major tumor suppressor in ccRCC. Miess et al. (237) discovered that exogenous overexpression of the VHL gene within cells can reduce intracellular lipid peroxides, thereby inhibiting the occurrence of ferroptosis. This study confirmed that VHL is an important gene in regulating ferroptosis sensitivity in ccRCC, demonstrating that VHL-induced ferroptosis could be a potential target for treating ccRCC. Mou et al. (238) used bioinformatics methods to discover that the expression of nuclear receptor coactivator 4 (NCOA4) is reduced in ccRCC and is associated with a poor prognosis in ccRCC patients. NCOA4 is closely linked to iron transport, and its decrease leads to a reduced sensitivity of tumor cells to ferroptosis. Therefore, promoting ferroptosis through targeting NCOA4 may be an effective method for treating ccRCC. However, this conclusion is solely based on bioinformatics analysis and may not be entirely reliable. Further experimental validation is needed to confirm its effectiveness. Artesunate (ART) is a chemical compound derived from the natural plant Artemisia annua, which has been shown to exhibit anti-tumor effects in various types of cancer (239). Markowitsch et al. found that ART can induce ferroptosis in drug-resistant kidney cancer cells, significantly inhibiting the progression of kidney cancer cells. This suggests that ART holds promise as an effective new drug for treating patients with drug-resistant renal cell carcinoma, addressing the issue of drug resistance in renal cancer (240).

Hereditary leiomyomatosis and renal cell cancer (HLRCC) is an autosomal dominant inherited disease that originates from germ-line mutations in the fumarate hydratase (FH) gene. It has a low incidence rate and is often solitary, but most cases present with metastasis at the time of diagnosis, with a median survival rate of 24 months (241). Despite some progress in understanding its pathogenesis, the primary treatment remains surgery, with relatively poor treatment outcomes. Therefore, the search for better treatment methods is crucial for extending the survival of patients. Michael et al. (242) found in their research that in hereditary leiomyomatosis and renal cell cancer cells, due to the inactivation of FH, GPX4 is succinated, leading to a decrease in its activity. Additionally, the activity of nuclear factor E2-related factor 2 (NRF2) increases, thereby preventing ferroptosis. Researchers suggest that future studies could explore NRF2 activity inhibitors applicable to humans or develop inhibitors targeting succinated GPX4 to induce ferroptosis in hereditary leiomyomatosis and renal cell cancer cells. This approach could lead to better treatment options for this disease and potentially open up new avenues for the precision treatment of renal cell cancer.

#### 4.5.1 The roles of iron metabolism and lipid metabolism in the progression of renal cell carcinoma

Iron accumulation is a main source of ROS, a crucial factor in ferroptosis. Increasing research indicates a close association between iron accumulation and the development of renal cell carcinoma, particularly ccRCC. Proteins involved in iron metabolism include ferritin light chain (FTL), ferritin heavy chain (FTH1), ferroportin (FPN), transferrin receptor 1 (TfR1) for iron uptake, and iron regulatory proteins 1 and 2 (IRP1/2) (243). Schnetz et al. found that genes related to iron metabolism are significantly upregulated in renal cell carcinoma tissues, especially in ccRCC tissues (243). In ccRCC tissues, the expression of FTL, FTH1, TfR1, and IRP1/2 proteins are upregulated, while the expression of FPN protein is downregulated, showing a phenotype of iron retention where iron accumulates within cancer cells. However, this iron accumulation does not trigger iron-induced cell death; instead, it promotes the development of renal cell carcinoma. FTH1 possesses ferroxidase activity, converting Fe^2+^ to Fe^3+^, which, upon binding with FTL, effectively reduces the toxicity of intracellular Fe^2+^, thus preventing cellular ferroptosis (244). Nuclear receptor coactivator 4 (NCOA4) is a component of the autophagosome involved in the process of ferritinophagy, the selective autophagy of ferritin proteins (245). Mou et al. analyzed the Cancer Genome Atlas (TCGA) database and found that NCOA4, a gene related to ferritinophagy, is closely associated with the malignancy and TNM staging of renal cell carcinoma (238). Research indicates that NCOA4 acts as a receptor for autophagy-related proteins 5 (ATG5) and 7(ATG7). The interaction between NCOA4 and ATG5/ATG7 promotes ferritinophagy, leading to a reduction in cellular ferritin levels, an increase in labile iron pools within cells, and ultimately triggering iron-dependent cell death in cancer cells. This indicates that the ATG5-ATG7-NCOA4 autophagy pathway may be a novel therapeutic target for treating renal cell carcinoma (92).

Lipid peroxidation is one of the main features of iron-dependent cell death, driving cells toward iron-induced death. However, ccRCC cells contain abundant lipid droplets but do not trigger iron-dependent cell death. The proportions of lipid components in lipid droplets vary, with PUFAs, particularly arachidonic acid and docosahexaenoic acid, playing important roles in the process of iron-dependent cell death (246). In ccRCC tissues, hypoxia-inducible factor2α (HIF-2α) can selectively enrich PUFAs through the mediation of lipid droplet-associated protein (HILPDA). ccRCC cells are not only more sensitive to iron-dependent cell death compared to normal kidney cells but also exhibit higher levels of PUFAs as the stage of ccRCC advances. The HIF-2α-HILPDA axis holds promise as a new therapeutic pathway for advanced kidney cancer. In certain ccRCC cells (such as FR1 and FR2 cells in the 786-O series), even with an increase in PUFA content, they do not exhibit increased sensitivity to iron-dependent cell death. Zou et al. (246) discovered that polyunsaturated ether phospholipids (PUFAePLs) can promote the evasion of iron-dependent cell death and increase sensitivity. The synthesis of PUFA-ePLs is associated with enzymes such as alkylglycerone phosphate synthase (AGPS) located in peroxisomes, fatty acyl-CoA reductase 1 (FAR1), glyceronephosphate O-acyltransferase (GNPAT), 1-acyl-sn-glycerol-3-phosphate acyltransferase 3 (AGPAT3) in the endoplasmic reticulum, and plasmalogen biosynthesis enzyme phytanoyl-CoA dihydroxyacetone phosphate acyltransferase (PEDS1). FR1 and FR2 cells can reduce PUFAePL levels by spontaneously downregulating AGPS, thereby decreasing cancer cell sensitivity to iron-dependent cell death, promoting cancer cell proliferation, and metastasis. Therefore, regulating cancer cell sensitivity to iron-dependent cell death by modulating AGPS expression can serve as a therapeutic strategy for treating iron-death-insensitive cancer cells. The transcription factor Nuclear factor erythroid 2–related factor 2 (Nrf2) is closely associated with iron-dependent cell death and is a member of the solute carrier family 7 member 11 (SLC7A11). It is also an upstream regulator of GPX4 and a key regulatory factor in cellular antioxidant responses. Nrf2 can protect cells from damage caused by lipid peroxidation products such as 4-hydroxynonenal and acrolein, thereby inhibiting iron-dependent cell death. Studies have indicated that the expression of Nrf2 and its pathway is associated with the staging and grading of kidney cancer, as well as resistance to targeted therapies and poor prognoses (247, 248). Therefore, Nrf2 could serve as a potential target for the treatment of advanced kidney cancer in the future. However, research on Nrf2 inhibitors, particularly in the context of kidney cancer, is currently limited. Currently discovered Nrf2 inhibitors include chlorobutanol in lung cancer research and berberine in head and neck cancer research. Both compounds can enhance tumor cell sensitivity to ferroptosis (249, 250). Further research is needed to determine whether the application of chlorobutanol or berberine in kidney cancer yields similar effects as seen in lung cancer and head and neck cancer, respectively.

#### 4.5.2 The role of the System X_c_^-^ GSH-GPX4 axis in the progression of kidney cancer

The System X_c_^-^ transports extracellular cystine into the cell and transports intracellular glutamate out of the cell to further GSH. GPX4 utilizes GSH to neutralize lipid peroxidation caused by ROS, thereby inhibiting ferroptosis in cells. Xu et al. (251) found that compared to normal kidney tissue, SLC7A11 is highly expressed in renal cancer tissue and inhibits ferroptosis by promoting GPX4 expression, thus promoting renal cancer cell proliferation, migration, and invasion. This indicates that SLC7A11 is one of the therapeutic targets for preventing the progression of renal cancer to metastatic renal cancer. GSH, as an essential intracellular antioxidant, can neutralize lipid peroxidation products. Gamma-glutamyltransferase 1 (GGT1) catalyzes the breakdown of extracellular GSH, providing cysteine for the generation of intracellular GSH, and is a component of the GSH recovery pathway. Bansal et al. (252) found that GGT1 levels are significantly increased in ccRCC cell lines (786-O and RCC10), and GGT1 can promote GSH synthesis, preventing ferroptosis caused by lipid peroxidation in tumor cells, thereby promoting tumor cell proliferation and metastasis. Studies have shown that the GGT1 inhibitor OU749 has minimal adverse effects and good efficacy, making it a promising new approach for treating ccRCC (253). Kruppel-like factor 2 (KLF2) is a member of the Kruppel-like factor family of transcription factors, characterized by a DNA-binding domain containing zinc fingers. Recent studies have found that KLF2 is involved in the development and progression of various cancers such as liver cancer, and kidney cancer, among others (254, 255). The downregulation of KLF2 expression is significantly correlated with the TNM staging of ccRCC, and ccRCC patients with low expression of KLF2 have significantly shorter overall survival and metastasis-free survival periods. In the study, a mouse model of ccRCC lung metastasis was developed, and it was found that compared to the low-expression group of KLF2, the group with KLF2 overexpression had smaller and significantly fewer lung metastatic nodules. Further research has revealed that KLF2 can bind to the promoter of GPX4 in ccRCC, leading to the downregulation of GPX4 expression, which protects ccRCC cells from ferroptosis, thus promoting tumor cell metastasis (255). Therefore, the System X_c_^-^GSH-GPX4 axis plays a crucial role in the progression of renal cancer and may represent a novel strategy for utilizing ferroptosis therapy in advanced renal cancer and even drug-resistant renal cancer in the future.

#### 4.5.3 The role of ferroptosis in the treatment of renal cancer

Ferroptosis is an emerging cancer suppression strategy, and identifying renal cancer patients who are sensitive to ferroptosis inducers quickly remains a challenging issue. 18F-TRX-PET can be used to predict the sensitivity of tumors to iron-targeted therapy, but its high cost limits its clinical applicability (256). *In situ* detection technology can assess the sensitivity of tumor tissues to ferroptosis. This technology involves using a high-power laser to induce local PUFA acyl chains in cell or tissue samples, generating lipid peroxidation, and demonstrating the sensitivity of cells or tissues to ferroptosis inducers on-site (257). Therefore, *in situ* detection is a cost-effective and convenient imaging technology that holds the promise of rapidly categorizing the sensitivity of cancer patients to ferroptosis. It could accelerate the development of targeted cancer therapies focusing on ferroptosis.

(1) Ferroptosis and immunotherapy in ccRCC

As the mechanism of ferroptosis continues to be elucidated, an increasing body of research suggests a close association between ferroptosis and the tumor microenvironment. NCOA4 is an autophagic component involved in the autophagic process of iron proteins FTH1 and FTL, capable of degrading iron proteins, releasing ferrous iron, and promoting cell ferroptosis. However, NCOA4 is generally underexpressed in ccRCC tissues. HU et al (258) discovered through the TCGA database that iron metabolism-related proteins FTH1 and FTL are upregulated in most solid tumor tissues. These proteins are associated with regulating T cells (Tregs) and tumor-associated macrophages (TAMs), especially M2 macrophages’ infiltration. Iron derived from M2 macrophages in TAMs can be exported through ferroportin (FPN) and secreted in the form of the iron carrier protein lipocalin-2 (LCN-2). This allows the transfer of iron to ccRCC cells, thereby promoting ccRCC cell proliferation. This correlation is positively associated with poor prognosis in patients. Treg cells are a major factor in creating an immunosuppressive tumor microenvironment. Infiltration of Treg cells in tumors is associated with higher pathological staging and poor prognosis in patients with ccRCC (259). Therefore, NCOA4 is a key molecule linking ferroptosis and immunotherapy. Studies indicate that the ferroptosis-related gene CARS is a potential immune-infiltration-related regulator of ferroptosis. Its high expression suggests a poor prognosis for patients and is positively correlated with PD-L1 expression in ccRCC. This indicates that CARS could be a potential target for immunotherapy in ccRCC (260). The above research indicates that iron metabolism and related genes in ferroptosis could serve as a point of entry for immunotherapy in ccRCC.

(2) Targeted therapy for ferroptosis in ccRCC

Targeted therapy is a frontline treatment for ccRCC with good clinical outcomes. However, with the continuous use of targeted drugs, some patients exhibit resistance. Ferroptosis, as a newly discovered modulated form of cell death, plays a significant role in the development and progression of kidney cancer. Previous studies have often highlighted the close relationship between ferroptosis and iron metabolism disturbance. However, recent research shows that zinc plays a role similar to iron in the process of ferroptosis (261). Zinc is transported between organelles and the cytoplasm through transport proteins in the SLC39 family (ZIP) or the SLC30 family (ZNT). Among these, ZIP7 participates in cell ferroptosis along with zinc. Lowering ZIP7 expression enables RCC4 cells to resist erastin-induced ferroptosis, but this protective effect can be eliminated by supplementing ZnCl2. This suggests that ZIP7 could serve as a potential therapeutic target in ccRCC (261). ccRCC tumors contain various subcellular lineages that exhibit different sensitivities to ferroptosis. How to enhance the sensitivity of these different cell lineages to ferroptosis, thereby improving the efficacy of ferroptosis inducers, remains a question that needs to be addressed. Research indicates that the susceptibility to ferroptosis is greatly influenced by cell density and fusogenicity. Cell density affects the sensitivity of cells to ferroptosis through the Hippo pathway. Further studies have revealed two compensatory downstream molecular pathways within the Hippo pathway: the Yes-associated protein (YAP) - S-phase kinase-associated protein 2 (SKP2) pathway and the WW domain-containing transcription regulator 1 (TAZ) - ERM protein 1 (EMP1) - NADPH oxidase 4 (NOX4) pathway. In renal cancer cells, the TAZ pathway is predominant (262). NOX4 can generate and accumulate superoxide and hydrogen peroxide, thereby promoting lipid peroxidation and the onset of ferroptosis. Upregulation of SKP2 can facilitate the expression of mRNA for serine/threonine kinases and transferrin receptors, thereby contributing to ferroptosis (263). It can be seen It is evident that the Hippo pathway mainly regulates ferroptosis through the involvement of SKP2 and NOX4, providing new insights for the treatment of ccRCC.

(3) Nanotherapy for ferroptosis in ccRCC

With the advancement of materials science, an increasing number of studies have found that nanoparticles can induce ferroptosis in various tumor cells including lung cancer, breast cancer, liver cancer, and others (264). Nanoparticles exhibit advantages such as improved solubility of small molecule ferroptosis inducers, enhanced targeting specificity, lower systemic toxicity, controllable drug release, and synergistic effects with emerging therapies. Nanoparticles have broad prospects in tumor treatment (265). A recent study discovered that using a peptide-modified iron oxide (Fe3O4) nanoformulation of 1 H-perfluorobutane (1 H-PFP) called GBP@Fe3O4 can trigger thermally induced ferroptosis in 786-O cells. Under 808 nm laser irradiation, localized moderate heat (45°C) triggered the liquid-gas transition of 1H-PFP, leading to the rapid release of Fe3O4 nanoparticles. This process generates a significant amount of ROS through the Fenton reaction in the tumor microenvironment. Meanwhile, heat stress reduces GSH synthesis, inhibiting the antioxidant response of tumor cells, and further exacerbating the damage caused by ROS. Simultaneously, tumor cells undergo lipid metabolism reprogramming, producing a large amount of lipid peroxides, ultimately leading to tumor-specific ferroptosis (266). This demonstrates that nanotechnology holds great promise as a potential treatment modality for ccRCC in the future. However, it currently remains in the early stages of basic research, with limited clinical studies and a need for further safety validation. In conclusion, the main mechanisms involved in ferroptosis—iron metabolism, lipid peroxidation, and the System X_c_^-^ GSH-GPX4 axis-are closely related to the progression of kidney cancer. They play vital roles in immunotherapy, targeted therapy, and potentially impactful nanotherapy for advanced kidney cancer. However, it remains unclear how ferroptosis regulates immune cell infiltration in the tumor microenvironment of kidney cancer and how it promotes the transition of M2 macrophages to M1 macrophages. Currently, ferroptosis inducers are still at the stage of cell and animal research due to their low solubility, weak targeting capabilities, and high systemic toxicity *in vivo*. With the advancement of nanotechnology, combining ferroptosis inducers with nanoparticles may help overcome the limitations of the inducers. However, there is limited research on their clinical application, and further investigation is required.

## 5 Prospects

Ferroptosis is a regulated form of cell death characterized by iron-catalyzed lipid peroxidation and can be modulated through various mechanisms. Extensive research has shown that ferroptosis plays a significant role in various cardiovascular diseases, degenerative diseases, and tumors, indicating the broad prospects for studying ferroptosis in CKD. In the regulation of ferroptosis through epigenetic modifications, only a few microRNAs are currently known to regulate the occurrence of ferroptosis. The role of other non-coding RNAs in this regulation remains unexplored. Current research on ferroptosis and kidney disease primarily focuses on acute and chronic renal conditions, particularly in the context of how ferroptosis contributes to the pathological processes of CKD such as interstitial fibrosis, mitochondrial dysfunction, inflammation, tubular cell regeneration, and other cellular processes. Ferroptosis may serve as a driver in converting maladaptive renal responses into CKD and represents a promising therapeutic target to halt disease progression. In the future, it will still be necessary to research specific biomarkers to identify ferroptosis *in vivo*, especially in the context of kidney disease pathology and its interaction with other modes of programmed cell death. As demonstrated in a recent study (267), metformin induces ferroptosis-mediated programmed cell death, exacerbating kidney damage. Neutrophils were identified as a significant trigger for metformin-induced renal toxicity. Specifically, the protein NGAL, in conjunction with iron and metformin, forms complexes that drive neutrophil infiltration into the kidneys, ultimately leading to NETosis and worsening AKI. In the future, concerning the research of ferroptosis inhibitors, current inhibitor studies are predominantly focused on animal research. Compounds like ferrostatin-1 and liproxstatin-1 have shown promising results in animal models, but further research is essential to ensure their safety and feasibility for human use. Due to the lack of clinical studies on some drugs, the efficacy of ferroptosis inhibitors in a clinical setting remains undetermined. Although some drugs have been used in clinical settings, several issues still need to be addressed in the future. For instance, paricalcitol regulates the antioxidant function of GPX4 by activating the vitamin D receptor, thereby inhibiting cisplatin-induced AKI (268). Iron chelators deferoxamine can alleviate ferroptosis and fibrosis in CKD rats (199). Antioxidants like vitamin E and melatonin also face similar challenges. Therefore, although numerous small molecule drugs have been discovered for inducing and inhibiting ferroptosis, these drugs have not yet been translated into clinical applications to benefit patients. We believe that future research can focus on the following aspects: 1) Further elucidating the mechanism of ferroptosis and identifying more regulators targeting crucial points of ferroptosis. 2) Continuing to investigate the relationship between ferroptosis regulation and kidney-related diseases, studying the role of ferroptosis in CKD. 3) Exploring the role of epigenetic modifications in mediating ferroptosis regulation. 4) Translating currently available small molecule drugs for regulating ferroptosis into clinical use. This review elaborates on the mechanism of ferroptosis and its research progress in kidney-related diseases, providing a solid foundation for the treatment, diagnosis, and related research of kidney-related diseases.

## Author contributions

ZL: Conceptualization, Validation, Writing – original draft. YL: Writing – review & editing. MY: Data curation, Formal analysis, Writing – original draft. XW: Formal analysis, Methodology, Writing – original draft. LZ: Writing – original draft. KY: Writing – review & editing.
